# Urinary Free Glycosaminoglycans Identify Adults at High Risk of Developing Early-stage High-grade Bladder Cancer

**DOI:** 10.1016/j.euros.2024.08.001

**Published:** 2024-08-23

**Authors:** Francesco Gatto, Sinisa Bratulic, Francesca Maccari, Fabio Galeotti, Nicola Volpi, Jens Nielsen, Yair Lotan, Henrik Kjölhede

**Affiliations:** a Department of Oncology-Pathology, Karolinska Institute, Stockholm, Sweden; b Department of Life Sciences, Chalmers University of Technology, Gothenburg, Sweden; c Elypta AB, Stockholm, Sweden; d Department of Life Sciences, University of Modena and Reggio Emilia, Modena, Italy; e BioInnovation Institute, Copenhagen, Denmark; f Department of Urology, UT Southwestern Medical Center at Dallas, Dallas, TX, USA; g Department of Urology, Sahlgrenska University Hospital, Gothenburg, Sweden; h Department of Urology, Institute of Clinical Science, Sahlgrenska Academy, University of Gothenburg, Gothenburg, Sweden

**Keywords:** Bladder cancer, Screening, Urinary biomarkers, Cancer metabolism, Glycosaminoglycans

## Abstract

**Background and objective:**

Screening for bladder cancer (BCa) could reduce mortality via early detection of early-stage high-grade (Ta/T1 N0 M0 grade 2–3) disease. Noninvasive biomarkers could aid in screening, but current markers lack the specificity required. The urinary free glycosaminoglycan profile (GAGome) is a promising biomarker for early detection of BCa metabolism.

**Methods:**

In a prospective case-control development study, we included patients with BCa or no evidence of disease (NED) and measured the urinary GAGome. We then developed a score to predict the probability of BCa using GAGome features that correlated with BCa versus NED according to Bayesian regression. Next, in a retrospective, population-based, case-control study, we included adults from the Lifelines Cohort Study who were presumed healthy at baseline. All cases with BCa confirmed in the cancer registry by the 2-yr or 6-yr study visit were matched to randomly selected control subjects. We developed a reference logistic regression model using age and sex to predict BCa at 7 yr after baseline. We then added the GAGome score to the model and assessed model improvement using the likelihood ratio test. We dichotomized outputs for the reference model and saturated model (reference + GAGome score) into high-risk versus low-risk categories using a 99% specificity cutoff and estimated the sensitivity for association with BCa at 7 yr.

**Key findings and limitations:**

We prospectively included 51 individuals with BCa and 38 with NED and observed alterations in three GAGome features compatible with BCa. We developed a score that discriminated BCa with an area under the receiver operating characteristic curve of 0.77 (95% confidence interval [CI] 0.67–0.87). We retrospectively selected a cohort of 1088 presumed healthy adults (median age 48 yr, 56% females), of whom 48 had developed BCa by 7 yr after baseline (median time to diagnosis 1.4 yr). The GAGome score was an independent predictor of BCa at 7 yr when added to the reference model (*p* < 0.001). The sensitivity for BCa at 7 yr for high-risk subjects was 31% (95% CI 20–43%) using the saturated model and 17% (95% CI 4.7–29%) using the reference model at 99% specificity (95% CI 98–99%).

**Conclusions and clinical implications:**

The urinary free GAGome is specifically altered in BCa and can be used for noninvasive identification of adults at high risk of developing BCa, independent of age and sex. This information could be useful for the design of risk-stratified targeted screening programs for BCa.

**Patient summary:**

We tested whether measurement of a class of sugars called glycosaminoglycans (GAGs) in urine could be used for early detection of bladder cancer. Our results show that GAG levels in urine can distinguish people at high risk of developing bladder cancer within 7 years, even if they are healthy at the time of the urine sampling.

## Take Home Message

In a prospective study, the urinary glycosaminoglycan profile (GAGome) was specifically altered in early-stage high-grade bladder cancer (BCa). In an independent retrospective cohort of otherwise healthy adults, a urinary GAGome BCa score identified subjects at high risk of developing BCa within 7 yr.

## 1 Introduction

Worldwide, bladder cancer (BCa) is responsible for ∼200 000 deaths annually [1]. Screening for BCa could reduce mortality by shifting the stage at diagnosis to early-stage high-grade disease (Ta/T1 N0 M0 and grade 2–3 [G2–3]) [2]. However, BCa prevalence in the average-risk population is too low to make cystoscopy (the reference standard for BCa diagnosis) cost effective [3]. While several noninvasive biomarkers have been approved for BCa detection [4], their specificity (60–85% [5]) would lead to excessive false-positive results when screening an average-risk population [6], [7]. Screening only populations at high risk of developing BCa, such as adults aged ≥50 yr with a significant smoking history and/or high-risk occupation, could overcome this limitation [7]. However, even for high-risk adults, the specificity should be ≥99% to render the diagnostic work-up feasible because the BCa prevalence can still be expected to be as low as 0.2% in this population, and at most ∼1.2% [8], [9]. Proposed risk models do not use any molecular biomarkers and instead rely on age, sex, tobacco use, and occupational exposure to define high risk [9], [10] and thus exclude a priori many demographic groups that could develop BCa. To the best of our knowledge, there are no validated molecular biomarkers to identify average-risk adults at high risk of developing BCa [11].

We previously sought to identify potential new biomarkers for cancer by analyzing the pan-cancer reprogramming of metabolism [12], a hallmark of cancer [13]. Our research identified glycosaminoglycans (GAGs), structurally diverse polysaccharides with complex sulfation and epimerization patterns implicated in tumor growth and invasion, as promising noninvasive biomarkers for renal cell carcinoma [14], [15], [16] and subsequently for 14 other cancer types [17], including BCa. In the latter study we estimated that the urinary free GAG profile (or GAGome) had 57% sensitivity and 95% specificity for BCa cases in comparison to healthy control subjects. However, the control subjects in the study were not representative of an average-risk population, with potential overestimation of the GAGome specificity. In addition, we did not estimate the sensitivity for early-stage high-grade BCa.

Here, we sought to establish if the urinary free GAGome could be a useful molecular biomarker of the risk of developing early stage high-grade BCa. To this end, we first conducted a prospective case-control development study to compare GAGomes between patients with bladder tumors undergoing transurethral resection of the bladder (TURB) and patients undergoing surveillance for bladder cancer with no evidence of disease (NED). Next, we carried out a retrospective population-based case-control study in adults aged ≥18 yr and presumed healthy at baseline with no history of cancer to establish whether the urinary free GAGome could predict the risk of developing BCa within 7 yr.

## 2 Patients and methods

### 2.1 Development study design

The development study is reported in compliance with the Standards for Reporting of Diagnostic Accuracy Studies (STARD) guidelines (Supplementary Text 1). Ethical permission was obtained from the Regional Ethical Review Committee of Gothenburg in November 2017 (#940-17). All study subjects provided signed informed consent. The study was registered on ClinicalTrials.gov as NCT05799456.

The study had a single-center, prospective, case-control diagnostic design. The protocol is included in the Supplementary material. Patients referred for TURB for BCa or for surveillance for recurrence after TURB formed two consecutive series. The inclusion criterion for cases was a primary BCa diagnosis at cystoscopy planned for TURB. The exclusion criterion was no histopathological diagnosis of BCa Ta–T4 N0–2 Mx–0 after TURB. For control subjects, the inclusion criteria were as follows: NED at cystoscopy assessed at least 6 mo after treatment with curative intent for Ta–T3 N0–2 M0 BCa or upper tract urothelial cancer; and no history of cancer apart from urothelial cancer. The exclusion criteria were positive cytology at the inclusion visit and any intravesical instillation therapy during the 6 mo before the inclusion visit.

At the pre-TURB visit for cases and at the follow-up visit for control subjects, patients provided an any-void urine sample, which was robotically centrifuged (2000 × *g* for 5 min at room temperature) and immediately stored in a secured access biobank at −80°C until analysis. Cystoscopy was performed in all patients (cases and control subjects) at the same clinic to assess the presence or absence of BCa as the reference standard using predefined standard-of-care diagnostic procedures.

### 2.2 Free GAGome measurements

Free GAGome analysis was performed at a single blinded central laboratory (Lablytica Life Sciences AB, Sweden) using a MIRAM GAGome kit (Product No FRUOV2, Elypta AB, Sweden). Concentrations (μg/ml) of 17 chondroitin sulfate (CS), heparan sulfate (HS), and hyaluronic acid (HA) disaccharides were detected and quantified using an ultra high-performance liquid chromatography-mass spectrometry/mass spectrometry (UHPLC-MS/MS) system (Acquity I-class Plus Xevo TQ-S micro, Waters Corporation, Milford, MA, USA) according to the instructions for use [18]. This assay was previously described [19] and the analytical performance characteristics and technical variability for urine samples were shown to be acceptable [18]. The assay omits proteolytic digestion, thereby theoratically limiting the quantified disaccharides to the protein-free fraction – hence free GAGomes. These 17 independent GAGome features were expanded to afford 18 additional dependent features: total CS and HS concentrations, and 16 CS or HS disaccharide mass fractions (μg/μg_Total_ as a percentage). The outliers were identified and excluded. Nondetectable features (<0.1 μg/ml in >50% patients) were not considered in downstream analyses. All samples from the patient population, including cases analyzed in our previous study [17] with first-generation (V1) kits, were reanalyzed using a single second-generation (V2) kit. We compared the performance of the V1 and V2 kits for each detectable urinary free GAGome feature in BCa patients analyzed as part of the previous versus the present study using a linear regression model.

### 2.3 Development of the urinary free GAGome BCa score

We developed a Bayesian logistic regression model to predict BCa versus NED (response variable) on the basis of detectable urinary free GAGome features as explanatory variables, using the projection predictive variable selection method [20]. In brief, a reference model was developed using all detectable GAGome features. Next, a “projected” model selected the minimal number of most informative GAGome features as final predictors using leave-one-out cross-validation to control for overfitting (internal validation). We normalized the output of the “projected” model in the range from 0 to 100 to define the urinary free GAGome BCa score (hereafter called the GAGome score for brevity). The model assumptions and description are reported in the Supplementary Text 2 [21]. The availability of data for the score is described in Section 2.7.

### 2.4 Diagnostic performance of the GAGome score

We assessed the discriminatory performance in terms of the area under the receiver operating characteristic curve (AUC) for classification of BCa versus NED with the GAGome score (index test) in comparison to BCa diagnosis via cystoscopy as the reference standard. The index test results were not available to the reference standard assessor, but not vice versa, as the reference standard results were required for development of the index test. There were no missing data. No indeterminate results were obtained. A predefined variability analysis of diagnostic accuracy was performed for the following subgroups: TaT1 N0 M0 G1, TaT1 N0 M0 G2–3, and T2a-4a N0-2 M0. The 95% confidence interval (CI) for the AUC was computed using bootstrap resampling (1000 bootstraps). Given that no prior estimates were known for the index test at the start of the study, we computed a sample size that would include >20 cases of G1 BCa (assumed to be reached by including 77 cases) and >25 controls. We estimated that the power for these sample sizes for detection of AUC > 0.75 for α < 0.05 was >99% [22].

### 2.5 Population-based study design

The population-based study is reported in compliance with the REMARK guidelines (Supplementary Text 3) and was a secondary analysis of the LEVANTIS-0087A study (ClinicalTrials.gov, NCT05235009). The LEVANTIS-0087A study was sponsored by Elypta AB (Stockholm, Sweden), which participated in the study design, execution, and analysis and in review of the final manuscript. Ethical permission was obtained from the Swedish Ethical Review Authority in October 2021 (#2021-04975), as amended in October 2023 (#2023-06519-02). The study had a retrospective, prospectively planned, population-based case-control design. The study population comprised adults aged ≥18 yr from the Lifelines Cohort Study [23] who were presumed healthy at baseline and had no history of cancer unless curatively treated >5 yr before baseline. Lifelines is a multidisciplinary, prospective, population-based cohort study examining the health and health-related behaviors of 167 729 individuals living in Northern Netherlands. All potential cases (ie, self-reported any type of cancer or death by the 2-yr or 6-yr study visit) in Lifelines were included and matched to randomly selected control subjects.

The inclusion criteria for cases were: age ≥18 yr, no diagnosis of cancer at the baseline visit or within 5 yr before the baseline visit unless curatively treated, and self-reported any type of cancer or death by the 2yr- or 6-yr study visit. The exclusion criterion was no confirmed diagnosis of BCa (ICD-10 topography code C67) according to linkage to the Dutch Cancer Registry. The inclusion criteria for controls were: age ≥18 yr, no diagnosis of cancer at the baseline visit or within 5 yr before the baseline visit unless curatively treated, and no self-reported cancer of any type by the 2-yr or 6-yr study visit. The exclusion criterion was a confirmed diagnosis of BCa (ICD-10 topography code C67) according to linkage to the Dutch Cancer Registry.

At the baseline visit, patients provided urine over a full 24-h period (22:00–22:00 h). The samples were shipped at room temperature to a central laboratory, robotically aliquoted, and kept refrigerated at 4°C before storage at −80°C (within ∼10 h after sample collection) until measurement of the urine GAGome, performed as described above blinded to the clinical data. Follow-up was performed for all subjects (cases and control subjects) via linkage to the Dutch Cancer Registry as the reference standard to assess the presence or absence of BCa after baseline, from the date of the baseline visit (between June 1, 2009 and March 1, 2016) up to December 31, 2021 or the date of death. Cases with a match in the registry after 6 yr from the baseline visit were not excluded (even if beyond the 6-yr study visit) up to 7 yr of follow-up.

### 2.6 Predictive performance of the GAGome score

We assessed the association between BCa within 7 yr after the baseline visit (response variable, binary) and age (modeled using linear tail-restricted cubic splines with 3 knots, continuous variable in years, 2 degrees of freedom [d.f.]), sex (binary, 1 d.f.), and the GAGome score (continuous variable with a probability score ranging from 0 to 100, 1 d.f.) as explanatory variables (predictors) using univariable logistic regression. No other explanatory variables were considered since we estimated a maximum of four degrees of freedom for model development (1 d.f. per 10 cases). Data on tobacco use were not collected. There were no missing data. Next, we developed two multivariable logistic regression models, a reference model using age and sex as predictors, and a saturated model to which we added the GAGome score as predictor to the reference model. The significance of the association between each predictor (including all nonlinear terms for age) and the response variable was assessed using the Wald test for meaningful changes in predictor values and is reported in terms of the odds ratio (OR) with 95% CI. Statistical significance was set at *p* < 0.05.

We determined an optimal cutoff for both the reference model and the saturated model to achieve the same clinically meaningful specificity level (99%), and dichotomized subjects as “high risk” versus “low risk”. We estimated the sensitivity for BCa versus no BCa within 7 yr after the baseline visit across the high-risk and low-risk groups in the overall population and across subgroups deemed useful in the design of a hypothetical screening program (age group, sex, tumor group at diagnosis, and time to diagnosis) by cross-tabulating the count of subjects with versus without BCa within 7 yr after the baseline versus against the count of subjects with high risk versus low risk.

We estimated that with a sample size of 46 cases and >1035 control subjects, an index test with 30% sensitivity and 99% specificity for BCa had power to detect marginal errors of 13% and 0.61%, respectively, at a confidence level of α < 0.05. All statistical analyses were conducted in *R* version 4.2.2 (R Foundation for Statistical Computing, Vienna, Austria).

### 2.7 Data availability

A synthetic data set with standardized GAGome values and the code used to develop the GAGome score via projection predictive variable selection will be deposited at https://github.com/SysBioChalmers/GAGome-BCa at the time of publication. The data used in the population-based study are accessible on request to the Lifelines Cohort Study. The data used in the development study are not publicly available because they involve clinical records protected by patient confidentiality. Requests for access to deidentified data can be directed to the corresponding author. Data that can be shared can be released via a data transfer agreement.

## 3 Results

### 3.1 Patient characteristics in the development study

Between October 2018 and February 2021, we prospectively enrolled 106 participants in the development study, of whom 57 elected for TURB for suspected BCa (cases) and 49 had NED after TURB for BCa or after nephroureterectomy for upper tract urothelial cancer (control subjects). After exclusions, we included 51 patients with pTa–4 N0–2 M0 BCa (three patients were excluded for non-BCa diagnosis and three because urine was not collected) and 38 with NED (six excluded because NED for <6 mo, three had positive cytology, one Tis, and one M1 BCa). The patient flow is shown in Supplementary Figure 1.

Baseline characteristics are shown in Table 1. The study population was generally typical of patients with newly diagnosed BCa or NED at cystoscopy after previous treatment for urothelial cancer. Other histopathological characteristics are shown in Supplementary Table 1. No adverse events were recorded during sample collection or cystoscopy evaluation.

**Table 1:** Baseline characteristics of individuals included in the development cohort (further characteristics in Supplementary Table 1)

	BCa (*n* = 51)	NED (*n* = 38)
Median age, yr (IQR)	75 (69–80)	73 (67–78)
Female, *n* (%)	11 (22)	13 (34)
History of non-BCa cancer, *n* (%)	5 (9.8)	0 (0)
pT stage, *n* (%)		
pTa	20 (39) b	29 (76) a
pT1	14 (28)	8 (21) a
pT2–4a	17 (33)	1 (2.6) a
Tumor grade, *n* (%)		
Grade 1	12 (23) b	17 (44.7) a
Grade 2	11 (22)	9 (23.7) a
Grade 3	28 (55)	12 (31.6) a
EAU prognostic risk group, *n* (%)		
Low-risk NMIBC	11 (22)	17 (45) a
Intermediate risk NMIBC	9 (18) b	5 (13) a
High risk NMIBC	14 (27)	15 (39) a
MIBC	17 (33)	1 (2.6) a
BCa tumor group, *n* (%)		
TaT1 N0 M0 grade 1	12 (24) b	0 (0%)
TaT1 N0 M0 grade 2–3	22 (43)	0 (0%)
T2a–4a N0–2 M0	17 (33)	0 (0%)
NED	0 (0)	38 (100%)
Median urinary free GAGome BCa score (IQR)	68 (52–76)	47 (37–58)

BCa = bladder cancer; EAU = European Association of Urology; IQR = interquartile range; NED = no evidence of disease; NMIBC = non–muscle-invasive bladder cancer; MIBC = muscle-invasive bladder cancer.

a Values refer to previous surgically treated BCa.

b One patient with missing pathological evaluation was classified on the basis of clinical findings.

### 3.2 Correlation between the urinary free GAGome and BCa diagnosis

We measured the free GAGome in 89 urine samples (one sample per patient) in a single-blinded central laboratory using a standardized UHPLC-MS/MS system. None of the samples were identified as outliers. We found that 17 of the 35 (49%) free GAGome features were detectable in this population (Supplementary Fig. 2). In comparison to our previous study that measured urinary free GAGomes with the V1 kit (in BCa cases only), results using the V2 kit revealed highly correlated concentrations for all detectable GAGome features (median *R*^2^ = 0.85), but much higher yields for HS and HA disaccharides (Supplementary Figs. 3 and 4).

Using Bayesian linear regression, we determined that three (18%) of the 17 detectable features were compatible with BCa versus NED (Supplementary Fig. 2 and Supplementary Table 2). Sensitivity analysis showed that estimates of the group differences were robust to prior choice (Supplementary Fig. 5). Specifically, BCa was compatible with an increase in total HA and a shift from *N-*sulfated HS to nonsulfated HS without a significant change in the total HS concentration in urine.

### 3.3 Development of the GAGome score BCa score for early detection of BCa

**Fig. 1:**
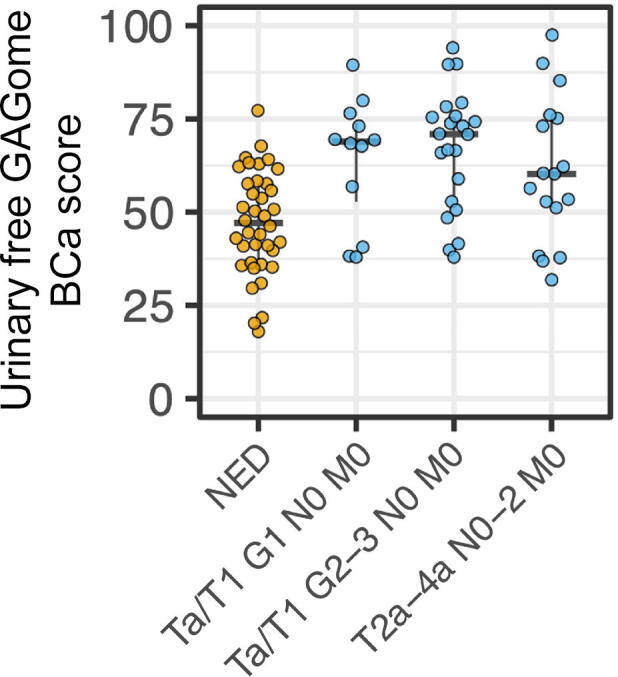
Urinary free GAGome bladder cancer (BCa) score in the development study for the cohort of 89 patients, of whom 51 had BCa (12 with Ta/T1 N0 M0 grade 1, 22 with Ta/T1 N0 M0 grade 2–3, and 17 with T2a–4a N0–2 M0) and 38 had no evidence of disease (NED). GAGome = glycosaminoglycan profile.

We developed a Bayesian logistic regression model to predict BCa versus NED using all detectable urinary free GAGome features as inputs. For the final model we selected two features as predictors: the total HA concentration and the *N-*sulfated HS mass fraction (Supplementary Table 3). Sensitivity analysis revealed that the choice of predictors was robust to different modeling assumptions (Supplementary Fig. 6). The model output (urinary free GAGome BCa score) was scaled between 0 and 100. In terms of discrimination between BCa and NED, the AUC was 0.77 (95% CI 0.67–0.87; Fig. 1 and Supplementary Table 4). In the Ta/T1 N0 M0 G2–3 subgroup (*n* = 60, 22 BCa), the AUC was 0.82 (95% CI 0.69–0.93). The performance for other subgroups is reported in Supplementary Table 4.

### 3.4 GAGome score performance in a population-based cohort

We retrospectively included 1088 participants from the Lifelines Cohort Study in a population-based cohort for analysis. Of these, 48 were diagnosed with BCa within 7 yr after their baseline visit (median time to diagnosis 1.4 yr).

Baseline characteristics are shown in Table 2, with additional characteristics in Supplementary Table 5. By design, the population was generally representative of the Lifelines Cohort Study [23]. Cases were generally older, more commonly male, and more likely to have hypertension than control subjects.

**Table 2:** Baseline characteristics in the population-based study (further characteristics in Supplementary Table 5)

	BCa cases (*n* = 48)	Control subjects (*n* = 1040)
Median age, yr (IQR)	61 (52–68)	47 (39–54)
Female, *n* (%)	16 (33)	594 (67)
Median follow-up, yr (IQR)	8.2 (6.8–11)	11 (7.8–11)
Time to diagnosis, *n* (%)		
0–1 yr	17 (35)	–
1–2 yr	17 (35)	–
2–4 yr	10 (21)	–
4–7 yr	<10 (<21)	–
No incident cancer	0 (0)	1040 (100)
BCa tumor group, *n* (%)		
Ta/T1 N0–x M0–x grade 1–x	<10 (<21)	–
TaT1 N0–x M0–x grade 2–3	31 (65)	–
T2a–4b N0–2 M0–1	<10 (<21)	–
Median urinary free GAGome BCa score (IQR)	56 (50–67)	49 (42–57)

BCa = bladder cancer; GAGome = glycosaminoglycan profile; IQR = interquartile range.

**Fig. 2:**
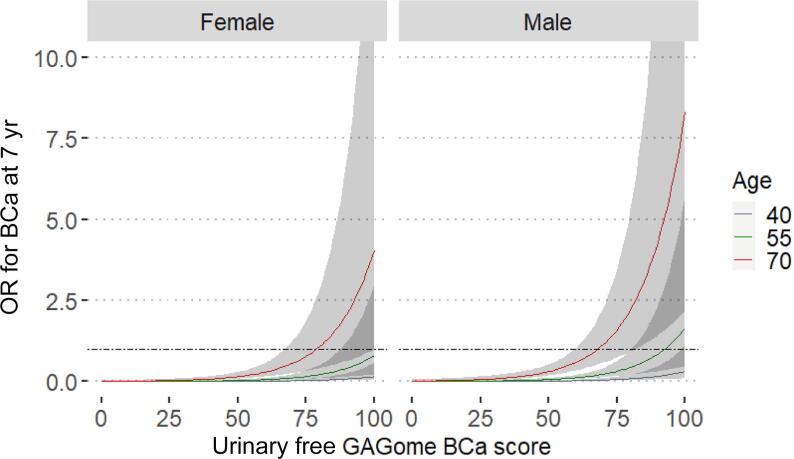
Predicted odds ratio (OR) for bladder cancer (BCa) at 7 yr after baseline given the urinary free GAGome BCa score in the saturated model for presumed healthy adults stratified by age at baseline and grouped by sex. The horizontal line represents OR = 1 and grey areas represent the 95% confidence interval. The *y*-axis is truncated at OR = 10 for legibility. GAGome = glycosaminoglycan profile.

The GAGome score was an independent predictor of BCa within 7 yr from baseline when added to the reference model, which uses only age and sex as predictors (likelihood ratio test *p* < 0.0001; Table 3 and Fig. 2). The GAGome score accounted for 29% of the variance explained by the saturated model. The AUC was 0.84 (95% CI 0.79–0.90) for the saturated model, 0.81 (95% CI 0.74–0.87) for the reference model, and 0.67 (95% CI 0.59–0.75) for the GAGome score alone.

**Table 3:** Association of BCa incidence by 7 yr with age, sex, and the urinary free GAGome BCa score measured at the baseline visit according to univariable and multivariable logistic regression^a^

Predictor	Univariable analysis		Multivariable analysis			
	OR (95% CI)	*p* value	Reference model		Saturated model	
			OR (95% CI)	*p* value	OR (95% CI)	*p* value
Age		<0.0001		<0.0001		<0.0001
40 yr	Reference		Reference		Reference	
55 yr	4.43 (2.14–9.19)		4.37 (2.08–9.19)		5.29 (2.44–11.5)	
70 yr	22.3 (9.34–53.1)		20.3 (8.42–48.8)		26.7 (10.6–67.4)	
Male sex (vs female)	2.66 (1.44–4.91)	0.002	2.21 (1.17–4.17)	0.015	2.06 (1.07–3.95)	0.030
Urinary free GAGome BCa score 70 (vs 50)	2.76 (1.75–4.36)	<0.0001	–		3.75 (2.20–6.42)	<0.0001

BCa = bladder cancer; GAGome = glycosaminoglycan profile; CI = confidence interval; OR = odds ratio

a Associations with continuous variables (age in years, and urinary free GAGome BCa score as probability on a scale from 0 to 100) are reported using clinically relevant values.

The sensitivity for BCa within 7 yr from baseline for adults classified as high risk was 31% (95% CI 20–43%) using the saturated model and 17% (95% CI 4.7–29%) using the reference model (Supplementary Table 6) at the same specificity (99%, 95% CI 98–99%). The performance of the saturated model was superior to that of the reference model across all subgroups (Supplementary Table 6). In particular, the sensitivity for high-risk adults in comparison to their low-risk counterparts using the saturated model versus the reference model was 19% (95% CI 0–41%) versus 0% (95% CI 0–0%) for females, 36% (95% CI 18–55%) versus 23% (95% CI: 10 - 38%) for Ta/T1 N0–x M0–x G2–3 disease, and 46% (95% CI 22–72%) versus 31% (95% CI 9.1–57%) for a BCa diagnosis within 1 yr from the baseline visit.

## 4 Discussion

We explored whether the urinary free GAGome could be a useful novel noninvasive biomarker of early-stage high-grade (Ta/T1 G2–3) BCa. Although we previously showed that BCa was correlated with an altered urinary GAGome in a fashion similar to that for other malignancies [17], the control subjects in our previous study were not fully representative of the spectrum of patients for whom biomarkers could be used to detect BCa, with possible overestimation of the specificity. Our results here confirm that the free GAGome is specifically altered in BCa, even in early-stage high-grade disease, and that this noninvasive biomarker is an independent risk factor for BCa in an average-risk population.

In comparison to our previous results, we observed some unexpected changes in the urinary GAGome attributable to BCa. We found an increase in the total HA concentration and a shift from *N*-sulfated HS to nonsulfated HS. We attribute these novel associations to the use of second-generation kits for GAG extraction, which have shown substantial increases in HA and HS yields in comparison to the first-generation kits used in our previous study. Previous research has investigated biofluidic GAGs in BCa on the basis of their ubiquity in the urothelial glycocalyx [24], a critical component of the protective bladder mucosal barrier [25]. None of these studies profiled the GAGome but rather focused on coarser urinary assays for total GAG determination. Nevertheless, these studies also reported a higher total HA concentration in BCa [26], [27], consistent with our findings. Previous studies have reported aberrations in unidentified urinary GAGs in BCa [22]. Our study confirmed and significantly expanded the role of urinary free GAGomes in the detection of early-stage high-grade BCa, whereby several structural aberrations appear to be specifically associated with the disease.

Several urinary biomarkers have been approved by regulatory authorities, although primarily for surveillance for BCa recurrence [5]. Despite their high sensitivity, their specificity is insufficient to control the false-positive rate in settings in which most subjects do not have BCa, as in screening programs. To the best of our knowledge, none of the urinary biomarkers approved for BCa were validated in a screening population [11]. The main strength of the urinary free GAGome appears to be its ability to detect a substantial proportion of BCa cases independent of other risk factors while retaining very high specificity, even in a population in which the prevalence of BCa could be expected to be as low as 10–20 per 100 000-person years [7]. We speculate that a risk model incorporating urinary free GAGomes could be used to implement a risk-stratified screening program in which only adults classified as “high risk” are screened. Our results indicate that just over one-third of incident early-stage high-grade BCa cases might be detected earlier using this approach. For comparison, the risk-stratified screening program for lung cancer recommended in the UK is estimated to exclude ∼40% of subjects who would develop lung cancer [28]. Despite this limitation, our proposed risk-stratified screening program would be more realistic to implement than an all-comer approach. According to our results, the urinary GAGome-based risk model would classify only ∼1% of all adults as “high risk”, substantially reducing the number of subjects to be screened.

Our study has some limitations. Given the rarity of BCa in an average-risk population, the sample size for BCa was small, leading to imprecise odds ratio and sensitivity estimates (particularly in the early-stage high-grade subgroup) and constraining the reference model to include only two risk factors as predictors. Notably, information on tobacco use was not collected and the independence of urinary free GAGomes could not be tested. Since the development study also included a sample size unlikely to span the full BCa disease spectrum, this limitation is not fully mitigated by the population-based study. Second, the retrospective case-control design might have excluded rare conditions that could alter urinary free GAGomes and affect the odds ratio and specificity; however, given the size of the control group, these potential conditions could be reasonably expected in <0.1% of the population. Despite these limitations, the validity of the urinary GAGome as a noninvasive biomarker appears to be well supported by its ability to predict BCa risk in an external population in our population-based analysis.

## 5 Conclusions

Our results show that urinary free GAGomes were specifically altered in BCa and were useful in predicting the risk of developing BCa in an external population independent of age and sex. This information could aid in the design of screening programs targeted at high-risk adults without excluding certain demographic groups a priori.

  ***Author contributions***: Francesco Gatto had full access to all the data in the study and takes responsibility for the integrity of the data and the accuracy of the data analysis.

  *Study concept and design*: Gatto, Kjölhede, Nielsen.

*Acquisition of data*: Kjölhede, Gatto, Volpi, Maccari, Galeotti.

*Analysis and interpretation of data*: Lotan, Gatto, Kjölhede.

*Drafting of the manuscript*: Gatto.

*Critical revision of the manuscript for important intellectual content*: All authors.

*Statistical analysis*: Gatto, Bratulic.

*Obtaining funding*: Gatto, Nielsen, Kjölhede.

*Administrative, technical, or material support*: None.

*Supervision*: Gatto, Kjölhede, Lotan.

*Other*: None.

  ***Financial disclosures:*** Francesco Gatto certifies that all conflicts of interest, including specific financial interests and relationships and affiliations relevant to the subject matter or materials discussed in the manuscript (eg, employment/affiliation, grants or funding, consultancies, honoraria, stock ownership or options, expert testimony, royalties, or patents filed, received, or pending), are the following: At the start of the study, Francesco Gatto and Jens Nielsen were listed as inventors on patent applications related to the biomarkers described in this study that were later assigned to Elypta AB. At the time of publication, Francesco Gatto and Jens Nielsen were shareholders in Elypta AB, Francesco Gatto and Sinisa Bratulic were employed at Elypta AB, and Jens Nielsen was a board member for Elypta AB. The remaining authors have nothing to disclose.

  ***Funding/Support and role of the sponsor*:** This study was financially supported by the grants from Knut and Alice Wallenberg Foundation (2017.0328 and 2018.0266), Cancerfonden (17.0625), and Ingabritt och Arne Lundbergs Forskningsstiftelse (2016.0011 and 2020.0023) to Chalmers University of Technology (Jens Nielsen) and by the Swedish state under an ALF agreement between the Swedish government and the county councils (ALFGBG-873181). The sponsors played a role in the design and conduct of the study; collection, management, analysis, and interpretation of the data; and preparation and review of the manuscript.

  ***Acknowledgments*:** This study was conducted using professional biobank services from Biobank West and Biobank Sweden. The Lifelines initiative was made possible by a subsidy from the Dutch Ministry of Health, Welfare and Sport, the Dutch Ministry of Economic Affairs, University Medical Center Groningen, Groningen University, and the Northern Netherlands provinces of Drenthe, Friesland, and Groningen.
