# Supplementary material for: Urinary Free Glycosaminoglycans Identify Adults at High Risk of Developing Early-stage High-grade Bladder Cancer
Source: Eur Urol Open Sci. 2024 Aug 23;68:40–7. doi: 10.1016/j.euros.2024.08.001 (PMC11387706; doi:10.1016/j.euros.2024.08.001)
Supplement: Supplementary Data 1 [file mmc1.pdf]

Revision History

Revision	Date	Author(s)	Description
1.0	11th October 2017	[redacted]	Created
1.1	17th October 2017	[redacted]	Fixes on Â§3.3. Defined end of follow-up period and some clinical variables.
1.2	11th January 2018	[redacted]	Changed biobank handling in sampling protocol.
1.3	2nd February 2018	[redacted]	Changed blood tube in sampling protocol.
2.0	3rd March 2020	[redacted]	Added new control arm. Elaborated eligibility criteria for controls. Removed exclusion criteria (prior history of cancer, history of radiation) and one inclusion criterion (available cytology results) for cases. Removed comparisons (all secondary endpoints) with cytology given its role in control eligibility criteria. Remove non-BCa subset analysis.

Glycosaminoglycan Profiling for Bladder Cancer Diagnostics

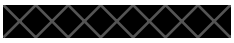

March 3, 2020

Contents

1 Introduction **3**

2 Study rationale **4**

2.1 Circulating biomarkers in BCa 4

2.2 Glycosaminoglycans as circulating cancer biomarkers 5

2.3 Objective of the study 6

3 Study design **8**

3.1 Study population 9

3.2 Eligibility criteria 9

3.3 Study data collection 9

3.4 Study endpoints 10

3.4.1 Primary endpoint 10

3.5 Study result presentation 11

3.6 Study logistics 11

3.6.1 Study period 11

3.6.2 Site of enrollment 11

3.6.3 Ethical considerations 11

3.6.4 Sample size 12

3.6.5 Expected patient flow 12

4 Protocols **13**

4.1 Sample collection protocol 13

Chapter 1

Introduction

The present document describes the protocol for a study to verify whether laboratory measurements of glycosaminoglycans (GAG) in plasma and/or urine samples can accurately distinguish bladder cancer (BCa) positive versus control subjects.
The present document contains the following items:

1. Study rationale;
2. Study design;
3. Protocols.

Chapter 2

Study rationale

This study aims at characterizing a novel class of circulating cancer biomarkers, namely glycosaminoglycans (GAG), in bladder cancer (BCa). Published evidence demonstrated the elevated diagnostic potential of circulating GAGs in clear cell renal cell carcinoma (ccRCC) with accuracy of detection versus healthy individuals ranging 92.7% to 100% depending whether only urine, only plasma, or both fluid combined were used. Preliminary results indicated a similar level of accuracy in prostate cancer (PCa). In this chapter, we will briefly describe the need of circulating biomarkers in cancer and BCa; the published and preliminary evidence of GAG as potential diagnostic biomarkers; the purpose of this study.

2.1 Circulating biomarkers in BCa

The current diagnostic workup of many cancers would largely benefit from simpler noninvasive or minimally invasive diagnostic procedures, for example a blood or urine test. Despite intense research, very few diagnostic tests based on blood or urine biomarkers entered clinical practice.

In BCa, early diagnosis is generally considered to produce a better outcome in patients. Contrary to muscle-invasive BCa (MIBC) or high-grade non-muscle invasive BCa (NMBIC), which tend to progress to MBIC, low-grade BCa is associated with generally good prognosis. In the asymptomatic population, the incidence of BCa is too low to warrant screening with current technologies (Roobol, M.J., et al. Feasibility study of screening for bladder cancer with urinary molecular markers (the BLU-P project). Urol Oncol, 2010. 28: 686.; Lotan, Y., et al. Should we screen for bladder cancer in a high-risk population?: A cost per life-year saved analysis. Cancer, 2006. 107: 982). In patients with hematuria, current urinary markers are not specific enough to replace cystoscopy, while urine cytology is not sensitive enough for low-grade BCa. Therefore, cystoscopy is the gold standard for primary diagnosis. The introduction of sensitive urinary biomarkers may reduce the need of unnecessary cystoscopy in symptomatic patients.

2.2 Glycosaminoglycans as circulating cancer biomarkers

One of the problems affecting the discovery of cancer-specific biomarkers in accessible fluids is the complex nature of cancer itself. Cancer heterogeneity remains a paramount challenge to develop effective biomarkers, in that future cancer biomarkers are required to be broad enough to capture the complexity of the cancer (sensitivity) - while narrow enough to exclude confounding diseases. A single molecule, e.g. a given protein, appears to have very limited biological potential to fulfill both requirements. A different way to tackle the problem of cancer heterogeneity is to rationalize a selected panel of biomarkers, broad enough to effectively speak for cancer while narrow enough to make its measurement amenable. We refer to such panels as systems biomarkers. A rational approach to discover systems biomarkers was put forward in the field of systems biology.

In a 2016 study, we utilized a systems biology approach to map omics data from clear cell renal cell carcinoma (ccRCC), the most common form of kidney cancer, onto an exhaustive reconstruction of the human metabolic network. The bioinformatics analysis eventually narrowed down the search to the pathway that leads to the synthesis and modification of GAGs, in particular on chondroitin and heparan sulfate. Virtually every step in this pathway was deregulated in ccRCC and exacerbated in metastasis. The candidate systems biomarker was therefore defined as the set of all possible modifications of these GAGs, a total of 18 independent GAG properties. A multicenter study was undertaken at Veneto Institute of Oncology in Padua, Italy and Sahlgrenska University Hospital in Gothenburg, Sweden to form a discovery cohort of retrospective and prospective samples from 50 healthy subjects and patients with metastatic ccRCC. GAGs were then measured in plasma and urine. The systems biomarker showed dramatic differences metastatic ccRCC versus healthy samples. We designed GAG scores to condense all the measurements that make up the systems biomarker. These GAG scores displayed an excellent ability to detect metastatic ccRCC with accuracies ranging 93.1% in the urine to 100% in the plasma. These figures were confirmed in an independent validation cohort comprising 33 new subjects, further demonstrating that the GAG scores normalize in patients with former diagnosis of ccRCC but no evidence of disease (NED). Results are collectively shown in Figure 2.1.

We have recently generated preliminary results that indicate that this systems biomarker could apply to PCa. Twenty-nine samples from patients with PCa collected at Prostate Cancer Center at the University of British Columbia in Vancouver, Canada were compared to 26 healthy individuals previously sampled. Samples were equally split into a discovery and validation cohort. We designed a blood and urine score that achieved exceptional accuracy in the discovery cohort. The validation of these scores in the validation cohort confirmed extremely elevated AUC, ranging 0.923 in the urine, 0.956 in the blood, and 0.995 when combined (Figure 2.2). Remarkably, the combined PCa score is specific to PCa as opposed to ccRCC (Figure 2.3). This indicates that GAGs acquire a specific signature in association with PCa, markedly distinct from

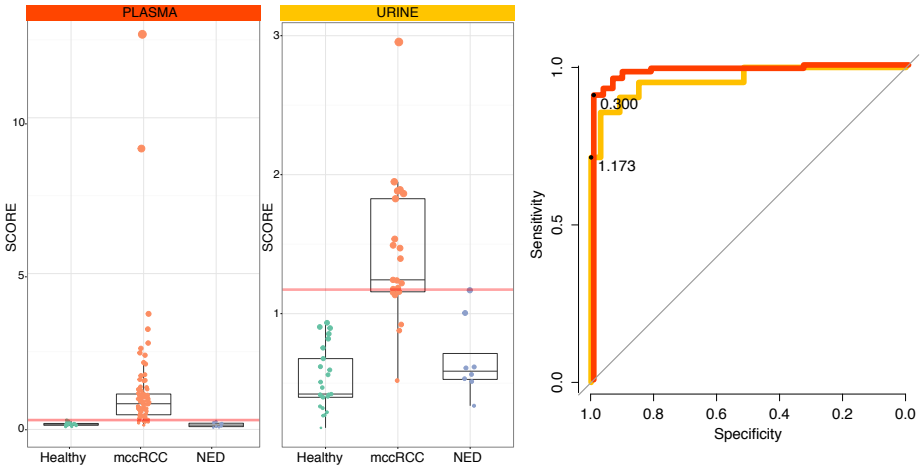

Figure 2.1: Plasma and urine score based on GAG profiling (left) and corresponding ROC curves (right) in healthy vs. metastatic clear cell renal cell carcinoma (mccRCC) vs. no evidence of disease (NED) subjects. Adapted from Gatto et al. (2016).

healthy individuals, and sufficiently different from ccRCC subjects.

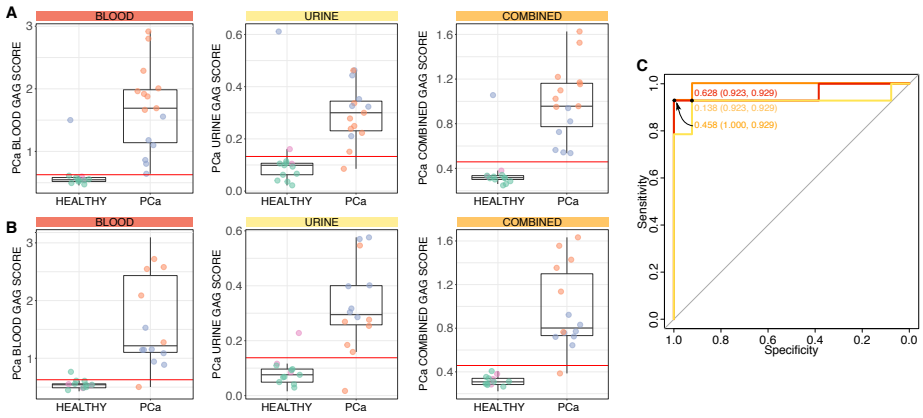

Figure 2.2: Plasma and urine score based on GAG profiling in the discovery (A) and validation (B) cohort of PCa vs. healthy samples. The ROC curves (C) refer to the validation cohort and the sensitivity/specificity at the optimal cutpoint found in the discovery cohort are displayed.

The results of these studies are suggestive that GAGs might be useful diagnostic biomarkers in urological malignancies. In this study, we will therefore evaluate the potential of GAG biomarkers in BCa.

2.3 Objective of the study

The overarching objective of this study is to verify whether laboratory measurements of glycosaminoglycans (GAG) in plasma and/or urine samples can

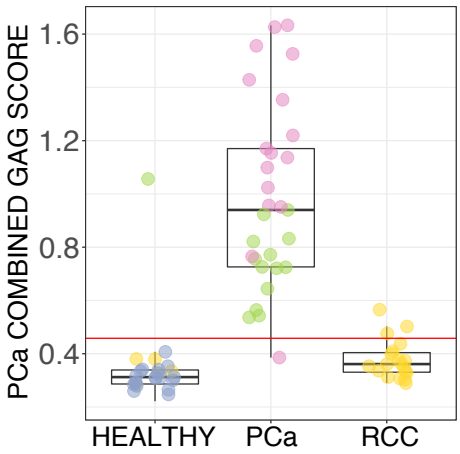

Figure 2.3: PCa combined score based on urine and blood GAG profiling in ccRCC vs. PCa vs. healthy samples.

accurately detect bladder cancer (BCa) in patients with symptoms suggestive of BCa.

The objective will be pursued by segmenting the study into 4 sequential phases with the following milestones:

1. Phase 1: Evaluation of GAG scores in a case-control study to distinguish BCa from control samples;
2. Phase 2: Estimation of operating characteristics of GAG scores in a prospective cohort study in BCa-positive versus BCA-negative subjects;
3. Phase 3: Estimation of operating characteristics of GAG scores in a prospective cohort study in the detection of BCa in symptomatic subjects;
4. Phase 4: Validation of sensitivity and specificity of GAG scores in a multicenter prospective cohort study in the detection of BCa in symptomatic subjects;

Chapter 3

Study design

The design of this study is articulated as a case-control study for phase 1, wherein continued enrollment in the case arm can constitute a cohort study for phase 2. A diagram of the study is shown in Figure 3.1.

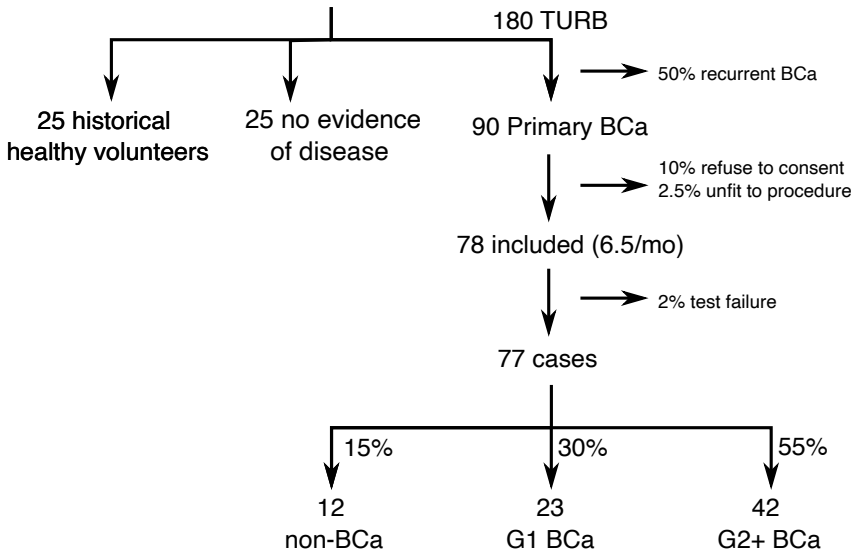

Figure 3.1: Study design. The study population is a consecutive series of patients referred to transurethral resection of the bladder (TURB) for BCa. In phase 1, a case-control study is formed by including healthy subjects as controls from a historical cohort and patients with no evidence of disease after TURB for BCa as clinical controls.

Table 3.1: Eligibility criteria in the case arm.

Inclusion criteria	Exclusion criteria
Referred to transurethral resection of the bladder (TURB) for primary BCa<br>Signed informed consent<br>Fit to undergo all protocol procedures	No histopathological diagnosis of BCa Ta-T4 N0-2 MX-0 after TURB<br>History of bladder or prostate radiation

Table 3.2: Eligibility criteria in the control arm for healthy volunteers.

Inclusion criteria	Exclusion criteria
$>$ 18 years of age<br>No diagnosis of cancer at the baseline visit or within 18 months after the baseline visit<br>Available glycosaminoglycans' measurements analysed in the same way as the prospective samples<br>Signed informed consent	Age- and sex-unmatched to cases

3.1 Study population

The study population is a consecutive series of patients referred to transurethral resection of the bladder (TURB) for BCa.

3.2 Eligibility criteria

For cases, the criteria were designed to obtain maximize a real-world distribution of significant BCa diagnoses as assessed by cystoscopy. The selection is biased towards higher grade BCa due to exclusion of cystoscopic findings treated by coagulation. The criteria are described in Table 3.1.

For controls, the criteria were different from historical healthy volunteers (Table 3.2) and patients with no evidence of disease (Table 3.3).

3.3 Study data collection

Each patient enrolled in this study will have two pieces of data collected - biological samples and clinical metadata. Biological samples collected for this study are described in Table 3.4. Clinical metadata collected for this study are

Table 3.3: Eligibility criteria in the control arm for patients with no evidence of disease.

Inclusion criteria	Exclusion criteria
In follow-up with cystoscopy after treatment Ta-T3 N0-2 M0 BCa or upper tract urothelial cancer and no evidence of disease at cystoscopy for at least 6 months prior to the inclusion visit	Positive cytology at the inclusion visit
No history of cancer except urothelial	Any intravesical instillation therapy during 6 months prior to the inclusion visit
Signed informed consent	

Table 3.4: Biological samples collected for each patient.

Sample type	Description
Plasma	12 x 220 uL of plasma from 1 blood draw is collected according to protocol in Chapter 4. It is collected pre-operatively.
Urine	12 x 220 uL of urine from 1 urine draw is collected according to protocol in Chapter 4. It is collected pre-operatively.

Table 3.5: Clinical metadata collected for each patient.

Variable	Description
Year of Birth	YYYY format
Family history of cancer	Yes/No and Relative (Father/Brother).
History of cancer	Yes/No and Histological Type
History of confounding morbidities	Yes/No for Urinary tract infection/Prostatitis/Prostate hyperplasia
Date of TURB	DD.MM.YYYY
Definitive diagnosis after TURB	BCa/Others
Urine cytology assessment of cancerous cells	Positive/Negative
Date of urine cytology	DD.MM.YYYY
Date of sampling	DD.MM.YYYY
Pathologic tumor grade	Tumor grade evaluated by pathologic diagnosis
Pathologic TNM stage	Tumor TNM stage evaluated by pathologic diagnosis
Number of tumors	Integer
Tumor diamater	In cm
Concurrent carcinoma in situ	Yes/No
Date of last known alive	DD.MM.YYYY as either last follow-up visit or per population registry
Status when last known alive	No evidence of disease/Localized/Locally-advanced/Metastatic/Unknown
Date of death	DD.MM.YYYY
Cause of death	BCa/Other/Unknown
Date of recurrence	DD.MM.YYYY as evaluated by cystoscopy
Date of progression	DD.MM.YYYY as evaluated by cystoscopy
Date of first cycle of intravesical treatment	DD.MM.YYYY
Type of intravesical treatment	Bacillus Calmette-Guerin/Mitomycin

described in Table 3.5. Follow-up data for a given patient will be collected up to 6 months after date of registration.

3.4 Study endpoints

3.4.1 Primary endpoint

The primary endpoint of the study is the AUC of GAG-scores in BCa vs. healthy controls. AUC is the area-under-the-receiver-operating-curve and GAG-scores are derived from a scoring system that uses measurements of GAGs in plasma and/or urine samples.

The following pre-specified subset analyses will be carried out:

1. Alternative case definitions:

   (a) Cases are defined as low-grade BCa;

3.5 Study result presentation

AUC is presented as the area under the receiver operating curve (ROC) for a given comparison. All case-control comparisons will be presented as 2x2 contingency tables. The unit of assessment is one subject.

An example of ROC and AUC calculation was provided in Figure 2.1 (right panel). An example of contingency table with definition of true positive (TP), true negative (TN), false positive (FP) and false negative (FN) is presented in Figure 3.2.

	Control	Case
Test+	FP	TP
Test-	TN	FN

Figure 3.2: Example of 2x2 contingency table.

3.6 Study logistics

This section describes the study period, the site of enrollments, ethical considerations, the sample size of the study, and the expected patient flow.

3.6.1 Study period

The study is estimated to last 36 months. Study start is contingent to ethical committee approval at the local Institutional Review Board. A tentative enrollment period is put forward between October 2018 and September 2021.

3.6.2 Site of enrollment

Patients will be recruited at Sahlgrenska Universitetssjukhuset (SU) by study Principal investigator [redacted] at the Department of Urology.

3.6.3 Ethical considerations

Ethical permit will be sought at the EtikprÃ¶vningsnÃ¤mnden (EPN) in GÃ¶teborg. Patient information to collect consent will be redacted in accordance to Good Clinical Practice and the Declaration of Helsinki.

3.6.4 Sample size

The study could not be powered because no prior knowledge on GAG scores in BCa was available at the time when it was designed. From prior studies in GAGs, it is known that a minimum sample size of 20 cases is required to observe a statistically signifincant signal versus healthy controls. We designed the study so that at least 20 G1 BCa were collected.

3.6.5 Expected patient flow

The expected flow of patients was represented in Figure 3.1. The flow was calculated based on estimates provided by the Department of Urology at SU.

Chapter 4

Protocols

There is one procedure used in this study, namely sample collection.

4.1 Sample collection protocol

Sampling must occur in an adequately clean and safe environment. Upon collection completion, samples are shipped to the Department of Biology and Biological Engineering at Chalmers University of Technology, Gothenburg, Sweden.

Plasma and blood collection

Blood sample per subjects is collected into 1 tubes with EDTA as anticoagulant. The tube for blood is a standard 8.5 mL EDTA vacuette for blood collection (for example K2E, K2 EDTA Greiner Bio-one nr 456243). The quantity of whole blood to be collected is 8.5 mL, enough to fill up a single sterile tube. Avoid having a tube sit at room temperature for more than 15 minutes. The tube is robotically centrifuged (2,000g 10 min at room temperature) and the plasma extracted and collected in 12 separate aliquots with 220 uL volume and immediately stored in a secured access biobank at -80Â°C.

Each tube must be marked/labelled with the corresponding sample ID. The sample ID is the composition of an arbitrary code generated by the recruitment center and the sampling date. Please use the ISO 8601 date format YYYY-MM-DD. Please mark the sample as *plasma* or *P* or *blood* or *B* depending on the fluid.

Urine collection

Urine sample per subject is collected in 1 cryovial tube VWR, Nr 479-0084 for up to 3.6 mL. The tube is robotically centrifuged (2,000g 5 min at room temperature) and the urine extracted and collected in 12 separate aliquots with 220 uL volume and immediately stored in a secured access biobank at -80Â°C.

Mark/label as above. Please mark the sample as *urine* or *U*.

Revision History

Revision	Date	Author(s)	Description
1.0	11th October 2017	[redacted]	Created
1.1	17th October 2017	[redacted]	Fixes on §3.3. Defined end of follow-up period and some clinical variables.
1.2	11th January 2018	[redacted]	Changed biobank handling in sampling protocol.
1.3	2nd February 2018	[redacted]	Changed blood tube in sampling protocol.

Glycosaminoglycan Profiling for Bladder Cancer Diagnostics

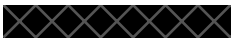

September 20, 2018

Contents

1 Introduction **3**

2 Study rationale **4**

2.1 Circulating biomarkers in BCa 4

2.2 Glycosaminoglycans as circulating cancer biomarkers 5

2.3 Objective of the study 6

3 Study design **8**

3.1 Study population 9

3.2 Eligibility criteria 9

3.3 Study data collection 9

3.4 Study endpoints 9

3.4.1 Primary endpoint 9

3.4.2 Secondary endpoints 10

3.5 Study result presentation 11

3.6 Study logistics 11

3.6.1 Study period 11

3.6.2 Site of enrollment 12

3.6.3 Ethical considerations 12

3.6.4 Sample size 12

3.6.5 Expected patient flow 12

4 Protocols **13**

4.1 Sample collection protocol 13

Chapter 1

Introduction

The present document describes the protocol for a study to verify whether laboratory measurements of glycosaminoglycans (GAG) in plasma and/or urine samples can accurately distinguish bladder cancer (BCa) positive versus control subjects.
The present document contains the following items:

1. Study rationale;
2. Study design;
3. Protocols.

Chapter 2

Study rationale

This study aims at characterizing a novel class of circulating cancer biomarkers, namely glycosaminoglycans (GAG), in bladder cancer (BCa). Published evidence demonstrated the elevated diagnostic potential of circulating GAGs in clear cell renal cell carcinoma (ccRCC) with accuracy of detection versus healthy individuals ranging 92.7% to 100% depending whether only urine, only plasma, or both fluid combined were used. Preliminary results indicated a similar level of accuracy in prostate cancer (PCa). In this chapter, we will briefly describe the need of circulating biomarkers in cancer and BCa; the published and preliminary evidence of GAG as potential diagnostic biomarkers; the purpose of this study.

2.1 Circulating biomarkers in BCa

The current diagnostic workup of many cancers would largely benefit from simpler noninvasive or minimally invasive diagnostic procedures, for example a blood or urine test. Despite intense research, very few diagnostic tests based on blood or urine biomarkers entered clinical practice.

In BCa, early diagnosis is generally considered to produce a better outcome in patients. Contrary to muscle-invasive BCa (MIBC) or high-grade non-muscle invasive BCa (NMBIC), which tend to progress to MBIC, low-grade BCa is associated with generally good prognosis. In the asymptomatic population, the incidence of BCa is too low to warrant screening with current technologies (Roobol, M.J., et al. Feasibility study of screening for bladder cancer with urinary molecular markers (the BLU-P project). Urol Oncol, 2010. 28: 686.; Lotan, Y., et al. Should we screen for bladder cancer in a high-risk population?: A cost per life-year saved analysis. Cancer, 2006. 107: 982). In patients with hematuria, current urinary markers are not specific enough to replace cystoscopy, while urine cytology is not sensitive enough for low-grade BCa. Therefore, cystoscopy is the gold standard for primary diagnosis. The introduction of sensitive urinary biomarkers may reduce the need of unnecessary cystoscopy in symptomatic patients.

2.2 Glycosaminoglycans as circulating cancer biomarkers

One of the problems affecting the discovery of cancer-specific biomarkers in accessible fluids is the complex nature of cancer itself. Cancer heterogeneity remains a paramount challenge to develop effective biomarkers, in that future cancer biomarkers are required to be broad enough to capture the complexity of the cancer (sensitivity) - while narrow enough to exclude confounding diseases. A single molecule, e.g. a given protein, appears to have very limited biological potential to fulfill both requirements. A different way to tackle the problem of cancer heterogeneity is to rationalize a selected panel of biomarkers, broad enough to effectively speak for cancer while narrow enough to make its measurement amenable. We refer to such panels as systems biomarkers. A rational approach to discover systems biomarkers was put forward in the field of systems biology.

In a 2016 study, we utilized a systems biology approach to map omics data from clear cell renal cell carcinoma (ccRCC), the most common form of kidney cancer, onto an exhaustive reconstruction of the human metabolic network. The bioinformatics analysis eventually narrowed down the search to the pathway that leads to the synthesis and modification of GAGs, in particular on chondroitin and heparan sulfate. Virtually every step in this pathway was deregulated in ccRCC and exacerbated in metastasis. The candidate systems biomarker was therefore defined as the set of all possible modifications of these GAGs, a total of 18 independent GAG properties. A multicenter study was undertaken at Veneto Institute of Oncology in Padua, Italy and Sahlgrenska University Hospital in Gothenburg, Sweden to form a discovery cohort of retrospective and prospective samples from 50 healthy subjects and patients with metastatic ccRCC. GAGs were then measured in plasma and urine. The systems biomarker showed dramatic differences metastatic ccRCC versus healthy samples. We designed GAG scores to condense all the measurements that make up the systems biomarker. These GAG scores displayed an excellent ability to detect metastatic ccRCC with accuracies ranging 93.1% in the urine to 100% in the plasma. These figures were confirmed in an independent validation cohort comprising 33 new subjects, further demonstrating that the GAG scores normalize in patients with former diagnosis of ccRCC but no evidence of disease (NED). Results are collectively shown in Figure 2.1.

We have recently generated preliminary results that indicate that this systems biomarker could apply to PCa. Twenty-nine samples from patients with PCa collected at Prostate Cancer Center at the University of British Columbia in Vancouver, Canada were compared to 26 healthy individuals previously sampled. Samples were equally split into a discovery and validation cohort. We designed a blood and urine score that achieved exceptional accuracy in the discovery cohort. The validation of these scores in the validation cohort confirmed extremely elevated AUC, ranging 0.923 in the urine, 0.956 in the blood, and 0.995 when combined (Figure 2.2). Remarkably, the combined PCa score is specific to PCa as opposed to ccRCC (Figure 2.3). This indicates that GAGs acquire a specific signature in association with PCa, markedly distinct from

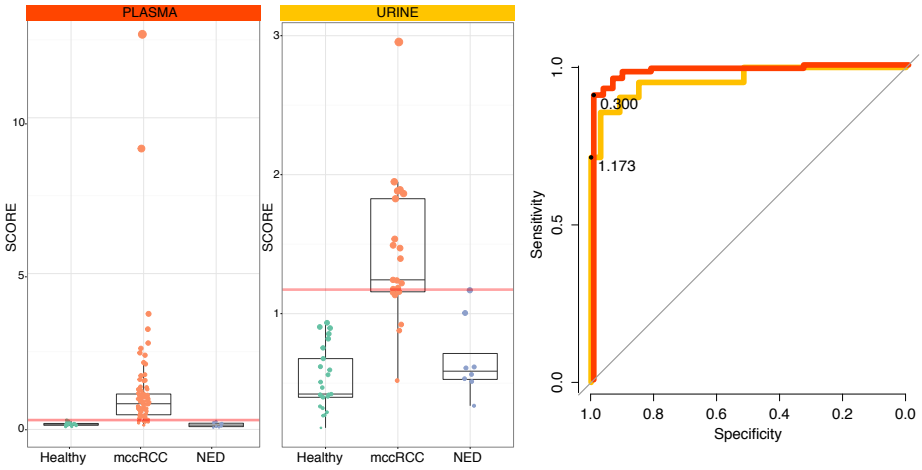

Figure 2.1: Plasma and urine score based on GAG profiling (left) and corresponding ROC curves (right) in healthy vs. metastatic clear cell renal cell carcinoma (mccRCC) vs. no evidence of disease (NED) subjects. Adapted from Gatto et al. (2016).

healthy individuals, and sufficiently different from ccRCC subjects.

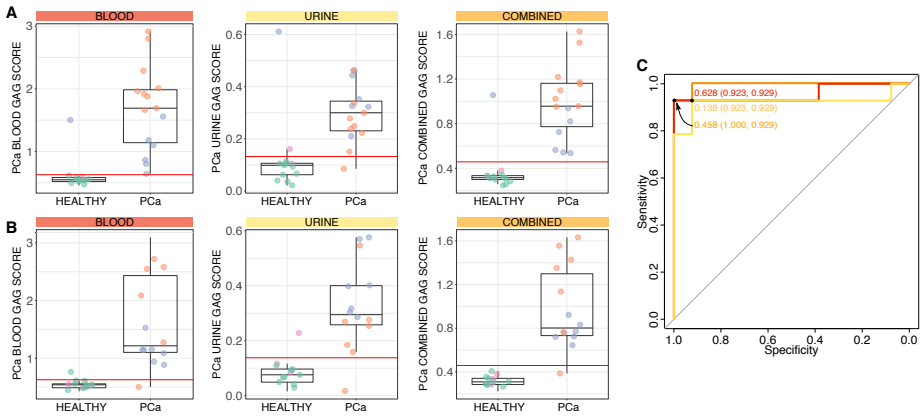

Figure 2.2: Plasma and urine score based on GAG profiling in the discovery (A) and validation (B) cohort of PCa vs. healthy samples. The ROC curves (C) refer to the validation cohort and the sensitivity/specificity at the optimal cutpoint found in the discovery cohort are displayed.

The results of these studies are suggestive that GAGs might be useful diagnostic biomarkers in urological malignancies. In this study, we will therefore evaluate the potential of GAG biomarkers in BCa.

2.3 Objective of the study

The overarching objective of this study is to verify whether laboratory measurements of glycosaminoglycans (GAG) in plasma and/or urine samples can

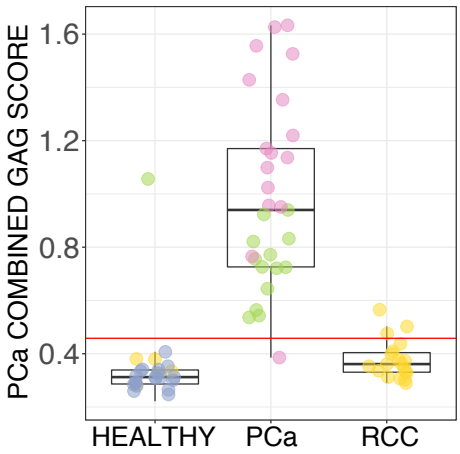

Figure 2.3: PCa combined score based on urine and blood GAG profiling in ccRCC vs. PCa vs. healthy samples.

accurately detect bladder cancer (BCa) in patients with symptoms suggestive of BCa.

The objective will be pursued by segmenting the study into 5 sequential phases with the following milestones:

1. Phase 1: Evaluation of GAG scores in a case-control study to distinguish BCa from healthy samples;
2. Phase 2: Estimation of operating characteristics of GAG scores in a prospective cohort study in BCa-positive versus BCA-negative subjects;
3. Phase 3: Differential operating characteristics of GAG scores vs. urine cytology in the detection of BCa and low-grade BCa from BCa-negative subjects;
4. Phase 4: Estimation of operating characteristics of GAG scores in a prospective cohort study in the detection of BCa in symptomatic subjects;
5. Phase 5: Validation of sensitivity and specificity of GAG scores in a multicenter prospective cohort study in the detection of BCa in symptomatic subjects;

Chapter 3

Study design

The design of this study is articulated as a case-control study for phase 1, wherein continued enrollment in the case arm can constitute a cohort study up to phase 3. A diagram of the study is shown in Figure 3.1.

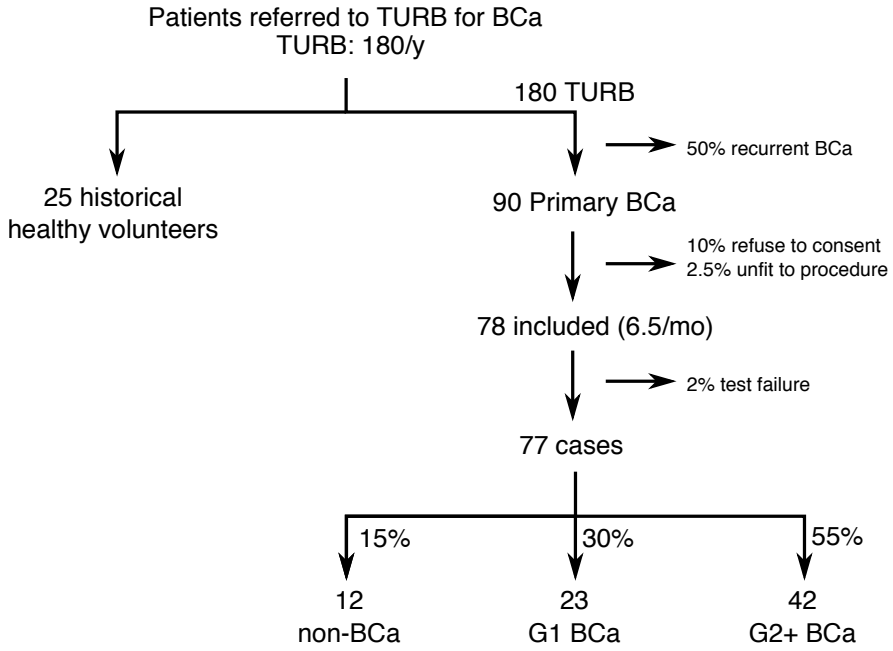

Figure 3.1: Study design. The study population is a consecutive series of patients referred to transurethral resection of the bladder (TURB) for BCa. In phase 1, a case-control study is formed by including healthy subjects as controls from a historical cohort

Table 3.1: Eligibility criteria in the case arm.

Inclusion criteria	Exclusion criteria
Referred to transurethral resection of the bladder (TURB) for BCa	Less than 18 years of age
Signed informed consent	History of bladder or prostate radiation
Urine cytology at diagnosis available	Prior diagnosis of cancer
Fit to undergo all protocol procedures	

Table 3.2: Biological samples collected for each patient.

Sample type	Description
Plasma	12 x 220 uL of plasma from 1 blood draw is collected according to protocol in Chapter 4. It is collected pre-operatively.
Urine	12 x 220 uL of urine from 1 urine draw is collected according to protocol in Chapter 4. It is collected pre-operatively.

3.1 Study population

The study population is a consecutive series of patients referred to transurethral resection of the bladder (TURB) for BCa.

3.2 Eligibility criteria

The eligibility criteria are defined only for the case arm of the study (see Figure 3.1). The criteria were designed to obtain maximize a real-world distribution of significant BCa diagnoses as assessed by cystoscopy. The selection is biased towards higher grade BCa due to exclusion of cystoscopic findings treated by coagulation. The criteria are described in Table 3.1.

3.3 Study data collection

Each patient enrolled in this study will have two pieces of data collected - biological samples and clinical metadata. Biological samples collected for this study are described in Table 3.2. Clinical metadata collected for this study are described in Table 3.3. Follow-up data for a given patient will be collected up to 6 months after date of registration.

3.4 Study endpoints

3.4.1 Primary endpoint

The primary endpoint of the study is the AUC of GAG-scores in BCa vs. healthy controls. AUC is the area-under-the-receiver-operating-curve and GAG-scores are derived from a scoring system that uses measurements of GAGs in plasma and/or urine samples.

Table 3.3: Clinical metadata collected for each patient.

Variable	Description
Year of Birth	YYYY format
Family history of cancer	Yes/No and Relative (Father/Brother).
History of cancer	Yes/No and Histological Type
History of confounding morbidities	Yes/No for Urinary tract infection/Prostatitis/Prostate hyperplasia
Date of TURB	DD.MM.YYYY
Definitive diagnosis after TURB	BCa/Others
Urine cytology assessment of cancerous cells	Positive/Negative
Date of urine cytology	DD.MM.YYYY
Date of sampling	DD.MM.YYYY
Pathologic tumor grade	Tumor grade evaluated by pathologic diagnosis
Pathologic TNM stage	Tumor TNM stage evaluated by pathologic diagnosis
Number of tumors	Integer
Tumor diamater	In cm
Concurrent carcinoma in situ	Yes/No
Date of last known alive	DD.MM.YYYY as either last follow-up visit or per population registry
Status when last known alive	No evidence of disease/Localized/Locally-advanced/Metastatic/Unknown
Date of death	DD.MM.YYYY
Cause of death	BCa/Other/Unknown
Date of recurrence	DD.MM.YYYY as evaluated by cystoscopy
Date of progression	DD.MM.YYYY as evaluated by cystoscopy
Date of first cycle of intravesical treatment	DD.MM.YYYY
Type of intravesical treatment	Bacillus Calmette-Guerin/Mitomycin

The following pre-specified subset analyses will be carried out:

1. Alternative case definitions:
   (a) Cases are defined as low-grade BCa;
2. Alternative control definitions
   (a) Controls are defined as all non-BCa in the case arm;

3.4.2 Secondary endpoints

The secondary endpoints revolve around different accuracy metrics than AUC and in a head-to-head comparison of GAG-scores to PSA testing. The following endpoints will be evaluated:

1. Specificity / sensitivity of GAG-scores for each case/control definition at optimal cut-offs for the GAG scores;
2. Positive / negative predictive value of GAG-scores in BCa vs. non-BCA at the optimal cut-off;
3. McNemar statistic for in sensitivity/specificity of GAG-scores vs. urine cytology in pre-operative samples in BCa vs. non-BCa at the optimal cut-offs;

4. Difference in positive / negative likelihood ratios of GAG-scores vs urine cytology in pre-operative samples in BCa vs. non-BCa at the optimal cut-offs;

5. McNemar statistic for in sensitivity/specificity of GAG-scores vs. urine cytology in pre-operative samples in low-grade BCa vs. non-BCa at the optimal cut-offs;

6. Difference in positive / negative likelihood ratios of GAG-scores vs urine cytology in pre-operative samples in low-grade BCa vs. non-BCa at the optimal cut-offs.

3.5 Study result presentation

AUC is presented as the area under the receiver operating curve (ROC) for a given comparison. All case-control comparisons will be presented as 2x2 contingency tables. The unit of assessment is one subject.

An example of ROC and AUC calculation was provided in Figure 2.1 (right panel). An example of contingency table with definition of true positive (TP), true negative (TN), false positive (FP) and false negative (FN) is presented in Figure 3.2.

	Control	Case
Test+	FP	TP
Test-	TN	FN

Figure 3.2: Example of 2x2 contingency table.

3.6 Study logistics

This section describes the study period, the site of enrollments, ethical considerations, the sample size of the study, and the expected patient flow.

3.6.1 Study period

The study is estimated to last 12 months. Study start is contingent to ethical committee approval at the local Institutional Review Board. A tentative enrollment period is put forward between December 2017 and November 2018.

3.6.2 Site of enrollment

Patients will be recruited at Sahlgrenska Universitetssjukhuset (SU) by study Principal investigator [redacted] at the Department of Urology.

3.6.3 Ethical considerations

Ethical permit will be sought at the Etikprövningsnämnden (EPN) in Göteborg. Patient information to collect consent will be redacted in accordance to Good Clinical Practice and the Declaration of Helsinki.

3.6.4 Sample size

The study could not be powered because no prior knowledge on GAG scores in BCa was available at the time when it was designed. From prior studies in GAGs, it is known that a minimum sample size of 20 cases is required to observe a statistically signifincant signal versus healthy controls. We designed the study so that at least 20 G1 BCa were collected.

3.6.5 Expected patient flow

The expected flow of patients was represented in Figure 3.1. The flow was calculated based on estimates provided by the Department of Urology at SU.

Chapter 4

Protocols

There is one procedure used in this study, namely sample collection.

4.1 Sample collection protocol

Sampling must occur in an adequately clean and safe environment. Upon collection completion, samples are shipped to the Department of Biology and Biological Engineering at Chalmers University of Technology, Gothenburg, Sweden.

Plasma and blood collection

Blood sample per subjects is collected into 1 tubes with EDTA as anticoagulant. The tube for blood is a standard 8.5 mL EDTA vacuette for blood collection (for example K2E, K2 EDTA Greiner Bio-one nr 456243). The quantity of whole blood to be collected is 8.5 mL, enough to fill up a single sterile tube. Avoid having a tube sit at room temperature for more than 15 minutes. The tube is robotically centrifuged (2,000g 10 min at room temperature) and the plasma extracted and collected in 12 separate aliquots with 220 uL volume and immediately stored in a secured access biobank at -80°C.

Each tube must be marked/labelled with the corresponding sample ID. The sample ID is the composition of an arbitrary code generated by the recruitment center and the sampling date. Please use the ISO 8601 date format YYYY-MM-DD. Please mark the sample as *plasma* or *P* or *blood* or *B* depending on the fluid.

Urine collection

Urine sample per subject is collected in 1 cryovial tube VWR, Nr 479-0084 for up to 3.6 mL. The tube is robotically centrifuged (2,000g 5 min at room temperature) and the urine extracted and collected in 12 separate aliquots with 220 uL volume and immediately stored in a secured access biobank at -80°C.

Mark/label as above. Please mark the sample as *urine* or *U*.
