# Supplementary material for: Urinary Free Glycosaminoglycans Identify Adults at High Risk of Developing Early-stage High-grade Bladder Cancer
Source: Eur Urol Open Sci. 2024 Aug 23;68:40–7. doi: 10.1016/j.euros.2024.08.001 (PMC11387706; doi:10.1016/j.euros.2024.08.001)
Supplement: Supplementary Data 2 [file mmc2.pdf]

Urinary Free Glycosaminoglycans Identify Adults at High Risk of Developing Early-Stage High-Grade Bladder Cancer

SUPPLEMENTARY MATERIAL

SUPPLEMENTARY TEXT

Supplementary Text 1. STARD checklist for the development study.

Section & Topic	No	Item	Reported on page #
**TITLE OR ABSTRACT**			
	**1**	Identification as a study of diagnostic accuracy using at least one measure of accuracy (such as sensitivity, specificity, predictive values, or AUC)	2
**ABSTRACT**			
	**2**	Structured summary of study design, methods, results, and conclusions (for specific guidance, see STARD for Abstracts)	2
**INTRODUCTION**			
	**3**	Scientific and clinical background, including the intended use and clinical role of the index test	3
	**4**	Study objectives and hypotheses	3
**METHODS**			
Study design	**5**	Whether data collection was planned before the index test and reference standard were performed (prospective study) or after (retrospective study)	4
Participants	**6**	Eligibility criteria	4
	**7**	On what basis potentially eligible participants were identified (such as symptoms, results from previous tests, inclusion in registry)	4
	**8**	Where and when potentially eligible participants were identified (setting, location and dates)	4, 7
	**9**	Whether participants formed a consecutive, random or convenience series	4
Test methods	**10a**	Index test, in sufficient detail to allow replication	4
	**10b**	Reference standard, in sufficient detail to allow replication	4
	**11**	Rationale for choosing the reference standard (if alternatives exist)	4
	**12a**	Definition of and rationale for test positivity cut-offs or result categories of the index test, distinguishing pre-specified from exploratory	Not applicable.
	**12b**	Definition of and rationale for test positivity cut-offs or result categories of the reference standard, distinguishing pre-specified from exploratory	5
	**13a**	Whether clinical information and reference standard results were available to the performers/readers of the index test	5
	**13b**	Whether clinical information and index test results were available to the assessors of the reference standard	5
Analysis	**14**	Methods for estimating or comparing measures of diagnostic accuracy	5
	**15**	How indeterminate index test or reference standard results were handled	5
	**16**	How missing data on the index test and reference standard were handled	5

	**17**	Any analyses of variability in diagnostic accuracy, distinguishing pre-specified from exploratory	5
	**18**	Intended sample size and how it was determined	5
**RESULTS**			
Participants	**19**	Flow of participants, using a diagram	Fig. S1
	**20**	Baseline demographic and clinical characteristics of participants	Table 1 and S1
	**21a**	Distribution of severity of disease in those with the target condition	Table 1 and S1
	**21b**	Distribution of alternative diagnoses in those without the target condition	Not applicable
	**22**	Time interval and any clinical interventions between index test and reference standard	4
Test results	**23**	Cross tabulation of the index test results (or their distribution) by the results of the reference standard	Not applicable
	**24**	Estimates of diagnostic accuracy and their precision (such as 95% confidence intervals)	9, Supplementary Table 4
	**25**	Any adverse events from performing the index test or the reference standard	7
**DISCUSSION**			
	**26**	Study limitations, including sources of potential bias, statistical uncertainty, and generalisability	15
	**27**	Implications for practice, including the intended use and clinical role of the index test	15
**OTHER INFORMATION**			
	**28**	Registration number and name of registry	4
	**29**	Where the full study protocol can be accessed	4
	**30**	Sources of funding and other support; role of funders	16

Supplementary Text 2. *Bayesian analysis reporting*. The goals of the analysis were to identify a minimal set of GAGome features which could reliably discriminate patients with bladder cancer (BCa) from no evidence of disease (NED). To this end, we fitted a Bayesian logistic model (estimated using MCMC sampling with 4 chains of 4000 iterations and a warmup of 2000) to predict the response (BCa vs NED) with Total CS [ug/mL], Total HS [ug/mL], Total HA [ug/mL], 0S CS [ug/mL], 4S CS [ug/mL], 6S CS [ug/mL], 4S6S CS [ug/mL], 2S6S CS [ug/mL], 0S CS [ug/ug %], 4S CS [ug/ug %], 6S CS [ug/ug %], 4S6S CS [ug/ug %], 2S6S CS [ug/ug %], 0S HS [ug/mL], NS HS [ug/mL], 0S HS [ug/ug %] and NS HS [ug/ug %] (formula: y ~ Total CS [ug/mL] + Total HS [ug/mL] + Total HA [ug/mL] + 0S CS [ug/mL] + 4S CS [ug/mL] + 6S CS [ug/mL] + 4S6S CS [ug/mL] + 2S6S CS [ug/mL] + 0S CS [ug/ug %] + 4S CS [ug/ug %] + 6S CS [ug/ug %] + 4S6S CS [ug/ug %] + 2S6S CS [ug/ug %] + 0S HS [ug/mL] + NS HS [ug/mL] + 0S HS [ug/ug %] + NS HS [ug/ug %]).

We specified prior distributions for the intercept and all the model parameters using *t*-student distributions centered around zero with a scale parameter of 2.5. The model's explanatory power was substantial (R^2 = 0.33, 95% CI [0.21, 0.43]). We then assessed the impact of prior distribution choices by repeating the procedure using both a narrower prior, using t-student distributions centered around zero with a scale parameter of 1.25, as well as a wider one, using t-student distributions centered around zero with a scale parameter of 5, for the intercept and all predictors (Supplementary Figure 6).

We reported the posterior distributions as the median of the posterior distribution and its 95% HDI (Highest Density Interval). Additionally, we used three key indices of effect sizes for each posterior distribution to summarize the effect's magnitude, direction, and practical relevance: a) probability of direction (*pd*, assessing the likelihood that the effect has a specific direction, e.g., positive or negative), b) probability of significance (i.e. the probability that *pd* exceeds a certain threshold), and c) the probability of a large effect (probability that the effect size exceeds predefined thresholds). The thresholds beyond which the effect was considered as having a direction, as being significant and as being large were 0, |0.09|, and |0.54|, respectively (Supplementary Table 3). Convergence and stability of the Bayesian sampling was assessed using R-hat, which should be below 1.01[1], and Effective Sample Size (ESS), which should be greater than 1000 [2]

The model's intercept, corresponding to Total CS [ug/mL] = 0, Total HS [ug/mL] = 0, Total HA [ug/mL] = 0, 0S CS [ug/mL] = 0, 4S CS [ug/mL] = 0, 6S CS [ug/mL] = 0, 4S6S CS [ug/mL] = 0, 2S6S CS [ug/mL] = 0, 0S CS [ug/ug %] = 0, 4S CS [ug/ug %] = 0, 6S CS [ug/ug %] = 0, 4S6S CS [ug/ug %] = 0, 2S6S CS [ug/ug %] = 0, 0S HS [ug/mL] = 0, NS HS [ug/mL] = 0, 0S HS [ug/ug %] = 0 and NS HS [ug/ug %] = 0, is at 0.57 (95% CI [0.04, 1.2]).

Within this model:

- The effect of Total CS [ug/mL] (Median = -681, 95% CI [-1758, 282]) has a 91% probability of being negative (< 0), 91% of being significant(< -0.09), and 91% of being

large (< -0.54). The estimation successfully converged (Rhat = 1.000) and the indices are reliable (Effective Sample Size (ESS) = 13265)

- The effect of Total HS [ug/mL] (Median = 28, 95% CI [-9.1, 67]) has a 93% probability of being positive (> 0), 93% of being significant (>0.09), and 93% of being large (> 0.54). The estimation successfully converged (Rhat = 1.000) and the indices are reliable (ESS = 14258)
- The effect of Total HA [ug/mL] (Median = 1.9, 95% CI [0.24, 4.0]) has a 99% probability of being positive (> 0), 99% of being significant (>0.09), and 94% of being large (> 0.54). The estimation successfully converged (Rhat = 1.000) and the indices are reliable (ESS = 13298)
- The effect of 0S CS [ug/mL] (Median = 350, 95% CI [-150, 909]) has a 91% probability of being positive (> 0), 91% of being significant (> 0.09), and 91% of being large (> 0.54). The estimation successfully converged (Rhat = 1.000) and the indices are reliable (ESS = 9540)
- The effect of 4S CS [ug/mL] (Median = 285, 95% CI [-112, 731]) has a 92% probability of being positive (> 0), 92% of being significant (> 0.09), and 92% of being large (> 0.54). The estimation successfully converged (Rhat = 1.000) and the indices are reliable (ESS = 13944)
- The effect of 6S CS [ug/mL] (Median = 68, 95% CI [-30, 176]) has a 91% probability of being positive (> 0), 91% of being significant (>0.09), and 91% of being large (> 0.54). The estimation successfully converged (Rhat = 1.000) and the indices are reliable (ESS = 13621)
- The effect of 4S6S CS [ug/mL] (Median = 13, 95% CI [-7.6, 36]) has a 89% probability of being positive (> 0), 89% of being significant (>0.09), and 88% of being large (> 0.54). The estimation successfully converged (Rhat = 1.000) and the indices are reliable (ESS = 13313)
- The effect of 2S6S CS [ug/mL] (Median = 7.7, 95% CI [-5.5, 22]) has a 87% probability of being positive (> 0), 87% of being significant (>0.09), and 85% of being large (> 0.54). The estimation successfully converged (Rhat = 1.000) and the indices are reliable (ESS = 13955)
- The effect of 0S CS [ug/ug %] (Median = -33, 95% CI [-104, 30]) has a 85% probability of being negative (< 0), 85% of being significant (<-0.09), and 85% of being large (< -0.54). The estimation successfully converged (Rhat = 1.000) and the indices are reliable (ESS = 13824)
- The effect of 4S CS [ug/ug %] (Median = -19, 95% CI [-59, 16]) has a 86% probability of being negative (< 0), 86% of being significant (< -0.09), and 85% of being large (< -0.54). The estimation successfully converged (Rhat = 1.000) and the indices are reliable (ESS = 13827)
- The effect of 6S CS [ug/ug %] (Median = -16, 95% CI [-49, 14]) has a 86% probability of being negative (< 0), 85% of being significant (< -0.09), and 85% of being large (< -0.54). The estimation successfully converged (Rhat = 1.000) and the indices are reliable (ESS = 13271)
- The effect of 4S6S CS [ug/ug %] (Median = -2.0, 95% CI [-7.5, 2.8]) has a 79% probability of being negative (< 0), 78% of being significant (< -0.09), and 72% of being large (< -

0.54). The estimation successfully converged (Rhat = 1.000) and the indices are reliable (ESS = 13569)

- The effect of 2S6S CS [ug/ug %] (Median = -2.0, 95% CI [-8.0, 3.3]) has a 77% probability of being negative (< 0), 76% of being significant (< -0.09), and 71% of being large (< -0.54). The estimation successfully converged (Rhat = 1.000) and the indices are reliable (ESS = 13275)
- The effect of 0S HS [ug/mL] (Median = -21, 95% CI [-53, 7.7]) has a 93% probability of being negative (< 0), 93% of being significant (< -0.09), and 93% of being large (< -0.54). The estimation successfully converged (Rhat = 1.000) and the indices are reliable (ESS = 14115)
- The effect of NS HS [ug/mL] (Median = -5.9, 95% CI [-15, 2.1]) has a 93% probability of being negative (< 0), 92% of being significant (< -0.09), and 91% of being large (< -0.54). The estimation successfully converged (Rhat = 1.000) and the indices are reliable (ESS = 13619)
- The effect of 0S HS [ug/ug %] (Median = 2.1, 95% CI [-0.4, 4.8]) has a 95% probability of being positive (> 0), 94% of being significant (> 0.09), and 89% of being large (> 0.54). The estimation successfully converged (Rhat = and the indices are reliable (ESS = 13509)
- The effect of NS HS [ug/ug %] (Median = 1.1, 95% CI [-12, 3.6]) has a 83% probability of being positive (> 0), 81% of being significant (> 0.09), and 69% of being large (> 0.54). The estimation successfully converged (Rhat = and the indices are reliable (ESS = 13278)

.

Supplementary Text 3. REMARK checklist for the population-based study.

	Item to be reported	Page no.
**INTRODUCTION**		
**1**	State the marker examined, the study objectives, and any pre-specified hypotheses.	**3**
**MATERIALS AND METHODS**		
Patients		
**2**	Describe the characteristics (e.g., disease stage or co-morbidities) of the study patients, including their source and inclusion and exclusion criteria.	**5**
**3**	Describe treatments received and how chosen (e.g., randomized or rule-based).	**NA**
Specimen characteristics		
**4**	Describe type of biological material used (including control samples) and methods of preservation and storage.	**6**
Assay methods		
**5**	Specify the assay method used and provide (or reference) a detailed protocol, including specific reagents or kits used, quality control procedures, reproducibility assessments, quantitation methods, and scoring and reporting protocols. Specify whether and how assays were performed blinded to the study endpoint.	**4, 6**
Study design		
**6**	State the method of case selection, including whether prospective or retrospective and whether stratification or matching (e.g., by stage of disease or age) was used. Specify the time period from which cases were taken, the end of the follow-up period, and the median follow-up time.	**5, 11**
**7**	Precisely define all clinical endpoints examined.	**6**
**8**	List all candidate variables initially examined or considered for inclusion in models.	**6**
**9**	Give rationale for sample size; if the study was designed to detect a specified effect size, give the target power and effect size.	**6**
Statistical analysis methods		
**10**	Specify all statistical methods, including details of any variable selection procedures and other model-building issues, how model assumptions were verified, and how missing data were handled.	**6**
**11**	Clarify how marker values were handled in the analyses; if relevant, describe methods used for cutpoint determination.	**6**
**RESULTS**		
Data		
**12**	Describe the flow of patients through the study, including the number of patients included in each stage of the analysis (a diagram may be helpful) and reasons for dropout. Specifically, both overall and for each subgroup extensively examined report the numbers of patients and the number of events.	**11**
**13**	Report distributions of basic demographic characteristics (at least age and sex), standard (disease-specific) prognostic variables, and tumor marker, including numbers of missing values.	**11**
Analysis and presentation		
**14**	Show the relation of the marker to standard prognostic variables.	**11**
**15**	Present univariable analyses showing the relation between the marker and outcome, with the estimated effect (e.g., hazard ratio and survival probability). Preferably provide similar analyses for all other variables being analyzed. For the effect of a tumor marker on a time-to-event outcome, a Kaplan-Meier plot is recommended.	**12**
**16**	For key multivariable analyses, report estimated effects (e.g., hazard ratio) with confidence intervals for the marker and, at least for the final model, all other variables in the model.	**12**

**17**	Among reported results, provide estimated effects with confidence intervals from an analysis in which the marker and standard prognostic variables are included, regardless of their statistical significance.	**12**
**18**	If done, report results of further investigations, such as checking assumptions, sensitivity analyses, and internal validation.	**NA**
**DISCUSSION**		
**19**	Interpret the results in the context of the pre-specified hypotheses and other relevant studies; include a discussion of limitations of the study.	**14**
**20**	Discuss implications for future research and clinical value.	**14**

SUPPLEMENTARY TABLES

Supplementary Table 1. Development study additional baseline characteristics. Continuous variables were summarized as median (interquartile range in brackets). Key: BCa – bladder cancer, IQR = interquartile range, NA – not applicable, NED – no evidence of disease.

	**BCa (N=51)**	**NED (N=38)**
**pN**		
N0	46 (90%)	0 (0%)*
N1-2	4 (7.8%)	0 (0%)*
Nx	1 (2.0%)**	38 (100%)*
**pM**		
M0	49 (96%)	0 (0%)*
Mx	2 (3.9%)**	38 (100%)*
**Tumor size (cm)**	2.5 (1.6, 3.4)**	NA*
**Number of tumors**	1.0 (1.0, 1.0)**	NA*
**Concurrent carcinoma in situ**	8 (16%)	0 (0%)

* Data on the previously surgically treated BCa was not collected for NED.
** One patient had missing pathologic evaluation. Baseline characteristics here reported were based on clinical findings.

Supplementary Table 2. Differences for BCa versus NED for each detectable urine free GAGome feature (in brackets, the 95% Credibility Interval [CI]). Free GAGome features were normalized by mean-centering and scaling by the standard deviation. Differences compatible with a change in a free GAGome features outside the Region of Practical Equivalence (ROPE) by <5% are highlighted in bold. Key: CS – chondroitin sulfate; HS – heparan sulfate; HA – hyaluronic acid.

**Free GAGome feature**	**Estimated group difference [95% CI]**	**Percentage of CI in ROPE**
**Total HA [ug/mL]**	**0.57 [0.15-0.99]**	**0%**
**0S HS [%]**	**0.53 [0.12-0.95]**	**0%**
0S HS [ug/mL]	0.31 [-0.10-0.72]	14%
Total HS [ug/mL]	0.29 [-0.13-0.71]	17%
6S CS [ug/mL]	0.28 [-0.13-0.69]	17%
4S6S CS [ug/mL]	0.28 [-0.13-0.69]	17%
6S CS [%]	0.25 [-0.17-0.68]	20%
2S6S CS [ug/mL]	0.23 [-0.18-0.65]	23%
4S6S CS [%]	0.22 [-0.19-0.64]	24%
4S CS [ug/mL]	0.20 [-0.22-0.62]	25%
4S CS [%]	0.18 [-0.24-0.59]	27%
2S6S CS [%]	0.17 [-0.25-0.59]	28%
Total CS [ug/mL]	0.14 [-0.29-0.57]	31%
NS HS [ug/mL]	0.13 [-0.30-0.55]	32%
0S CS [ug/mL]	0.05 [-0.38-0.46]	38%
0S CS [%]	-0.24 [-0.67-0.18]	20%
**NS HS** [%]	**-0.61 [-1.01--0.20]**	**0%**

Supplementary Table 3. Median of the posterior distribution and its 95% HDI (Highest Density Interval) for each predictor in the "full" model, and assessment of the probability that the posterior had a direction, was significant, or was large. The features selected as predictors for the final model were highlighted in bold. Key: HDI – Highest Density Interval.

**Parameter**	**Median**	**HDI interval**	**HDI lower bound**	**HDI upper bound**	**Probability of Direction**	**Probability of Significance**	**Probability of Being Large**
(Intercept)	0.57	0.95	0.04	1.2	0.98	0.96	0.54
Total CS [ug/mL]	−681	0.95	−1,757	282	0.91	0.91	0.91
Total HS [ug/mL]	28	0.95	−9.1	67	0.93	0.93	0.93
**Total HA [ug/mL]**	**1.9**	**0.95**	**0.24**	**4.0**	**0.99**	**0.99**	**0.94**
0S CS [ug/mL]	350	0.95	−150	909	0.91	0.91	0.91
4S CS [ug/mL]	285	0.95	−112	731	0.92	0.92	0.92
6S CS [ug/mL]	68	0.95	−30	176	0.91	0.91	0.91
4S6S CS [ug/mL]	13	0.95	−7.6	36	0.89	0.89	0.88
2S6S CS [ug/mL]	7.8	0.95	−5.5	22	0.87	0.87	0.85
0S CS [%]	−32	0.95	−104	30	0.85	0.85	0.85
4S CS [%]	−19	0.95	−59	16	0.86	0.86	0.85
6S CS [%]	−16	0.95	−49	13	0.86	0.85	0.85
4S6S CS [%]	−2.0	0.95	−7.5	2.8	0.80	0.79	0.72
2S6S CS [%]	−2.0	0.95	−8.0	3.3	0.77	0.76	0.71
0S HS [ug/mL]	−21	0.95	−53	7.7	0.93	0.93	0.93
NS HS [ug/mL]	−5.9	0.95	−13	2.1	0.93	0.92	0.91
0S HS [%]	2.1	0.95	−0.39	4.8	0.95	0.94	0.89
**NS HS [%]**	**1.1**	**0.95**	**−1.2**	**3.6**	**0.83**	**0.81**	**0.69**

Supplementary Table 4. AUC for the classification of BCa vs. NED using the urine free GAGome BCa score in the development study population and by subset: TaT1 N0 M0 G1 vs. TaT1 N0 M0 G2-3 vs. T2a-4a N0-2 M0. In brackets, the 95% confidence interval.

Subset	AUC
All (N = 89, 51 BCa)	0.77 (0.67 to 0.87)
TaT1 N0 M0 G1 (N = 50, 12 BCa)	0.78 (0.59 to 0.95)
TaT1 N0 M0 G2-3 (N = 60, 22 BCa)	0.82 (0.69 to 0.93)
T2a-4a N0-2 M0 (N = 55, 17 BCa)	0.70 (0.54 to 0.85)

Supplementary Table 5. Population-based study additional baseline characteristics. Continuous variables were summarized as median (interquartile range in brackets). Key: BCa – bladder cancer, BMI – body mass index, CRP – C-reactive protein, HDL – high-density lipoprotein.

	Cases (N=48)	Controls (N=1040)
**BMI (kg/m2)**	26 [24, 29]	26 [23, 28]
**Has hypertension**	21 (44%)	255 (25%)
**Diabetes**	0 (0%)	37 (3.6%)
**Cancer type (ICD 10)**		
C67.0	<10 (<21%)	0 (0%)
C67.1	<10 (<21%)	0 (0%)
C67.2	12 (25%)	0 (0%)
C67.3	<10 (<21%)	0 (0%)
C67.4	<10 (<21%)	0 (0%)
C67.5	<10 (<21%)	0 (0%)
C67.6	<10 (<21%)	0 (0%)
C67.8	14 (29%)	0 (0%)
C67.9	<10 (<21%)	0 (0%)
Not applicable	0 (0%)	1040 (100%)
**TNM stage**		
Stage 0	27 (56%)	0 (0%)
Stage 1	13 (27%)	0 (0%)
Stage 2-4	<10 (<21%)	0 (0%)
Not applicable	0 (0%)	1040 (100%)
**Tumor grade**		
G1	<10 (<21%)	0 (0%)
G2	16 (33%)	0 (0%)
G3	21 (44%)	0 (0%)
GX	<10 (<21%)	0 (0%)
Not applicable	0 (0%)	1040 (100%)
**Serum CRP [mg/L]**	1.0 [0.75, 1.8]*	1.0 [0.54, 2.4]**
**Serum HDL [mmol/L]**	1.4 [1.2, 1.8]	1.4 [1.2, 1.7]
**Serum hemoglobin [mmol/L]**	9.0 [8.4, 9.4]***	8.8 [8.3, 9.3]
**Urine creatinine [mmol/L]**	7.4 [5.1, 10]	7.5 [5.2, 11]

* 30 (63%) missing data.
** 364 (35%) missing data.
*** 1(2.1%) missing datum.

Supplementary Table 6. Sensitivity and specificity for BCa in 7 years after baseline in adults classified as "High risk" vs. "Low risk" according to the reference vs. saturated model in the population-based study (*N* = 1088, 1040 controls vs 48 cases) and across subsets. In brackets, the 95% confidence interval (CI).

				Reference model		Saturated model	
**Subsets**	**N**	**N cases**	**N controls**	**Sensitivity [95% CI]**	**Specificity [95% CI]**	**Sensitivity [95% CI]**	**Specificity [95% CI]**
**All**	1088 (100%)	48 (100%)	1040 (100%)	17% [4.7%, 29%]	99% [98%, 99%]	31% [20%, 43%]	99% [98%,99%]
**Sex**							
Males	478 (44%)	32 (67%)	446 (43%)	25% [12%, 41%]	97% [96%, 98%]	38% [18%, 53%]	98% [96% , 99%]
Females	610 (56%)	16 (33%)	594 (57%)	0% [0%, 0%]	100% [99%, 100%]	19% [0%, 41%]	99% [99%, 100%]
**Age**							
< 50 y/o	635 (58%)	<10 (<21%)	627 (60%)	0% [0%, 0%]	99% [99%, 100%]	12% [0%, 40%]	100% [100%, 100%]
50 to 69 y/o	425 (39%)	32 (67%)	393 (38%)	9.4% [0%, 20%]	99% [98%, 100%]	22% [8.8%, 40%]	98% [96%, 100%]
> 70 y/o	28 (2.6%)	<10 (<21%)	20 (1.9%)	62% [20%, 100%]	45% [24%, 67%]	100% [100%, 100%]	60% [40%, 81%]
**BCa tumor group**							
TaT1 N0X M0X G1	1049 (96%)	<10 (<21%)	1040 (100%)	0% [0%, 0%]	99% [98%, 99%]	11% [0%, 40%]	98% [98%, 99%]
TaT1 N0X M0X G2-3	1071 (98%)	31 (65%)	1040 (100%)	23% [10%, 38%]	99% [98%, 99%]	36% [18%, 55%]	99% [98%, 99%]
T2A-4B N0-2 M0-1	1048 (96%)	<10 (<21%)	1040 (100%)	12% [0%, 33%]	99% [98%, 99%]	38% [0%, 83%]	98% [98%, 99%]
**Time to diagnosis**							
0 to <1 year	1053 (97%)	13 (27%)	1040 (100%)	31% [9.1%, 57%]	99% [98%, 99%]	46% [22%, 72%]	98% [98%, 99%]
0 to <3 years	1077 (99%)	37 (77%)	1040 (100%)	16% [6.1%, 28%]	99% [98%, 99%]	32% [16%, 48%]	99% [98%, 99%]

SUPPLEMENTARY FIGURES

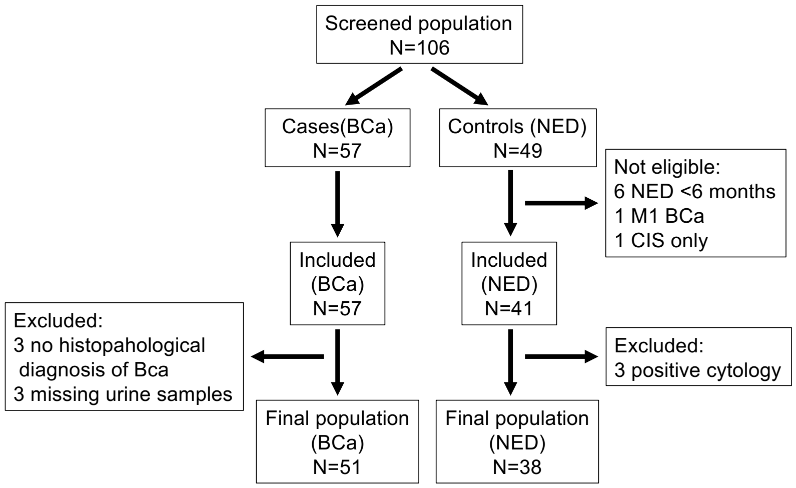

Supplementary Figure 1. Patient flow in the development study.

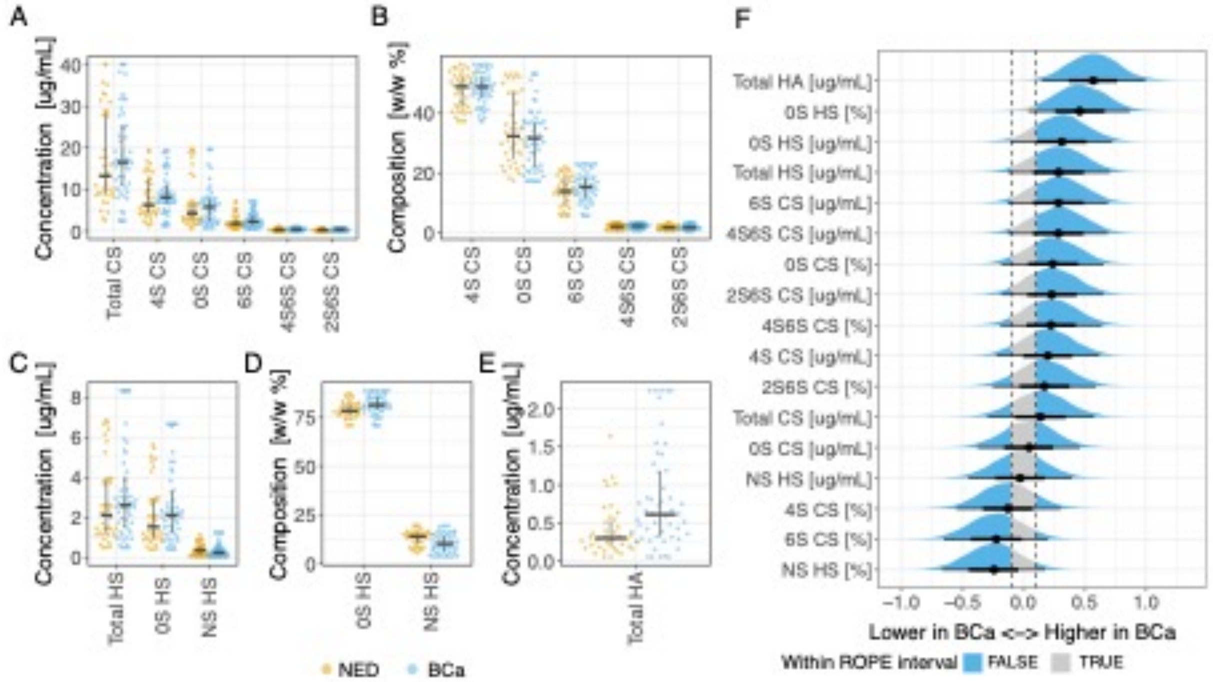

Supplementary Figure 2. A-E) Urine free GAGome per group, with 90% winsorization. **F)** Correlation of 17 detectable urine free GAGome features in 89 patients with BCa (*N* = 51) vs. NED (*N* = 38). The region of practical equivalence (ROPE) was marked by the two vertical dashed lines. A free GAGome feature was deemed compatible with BCa or NED if its 95% credible interval (CI) did not fall inside the ROPE by >5%. Key: CS – chondroitin sulfate, HS – heparan sulfate, HA – hyaluronic acid, BCa – bladder cancer, NED – no evidence of disease, ROPE – region of practical equivalence.

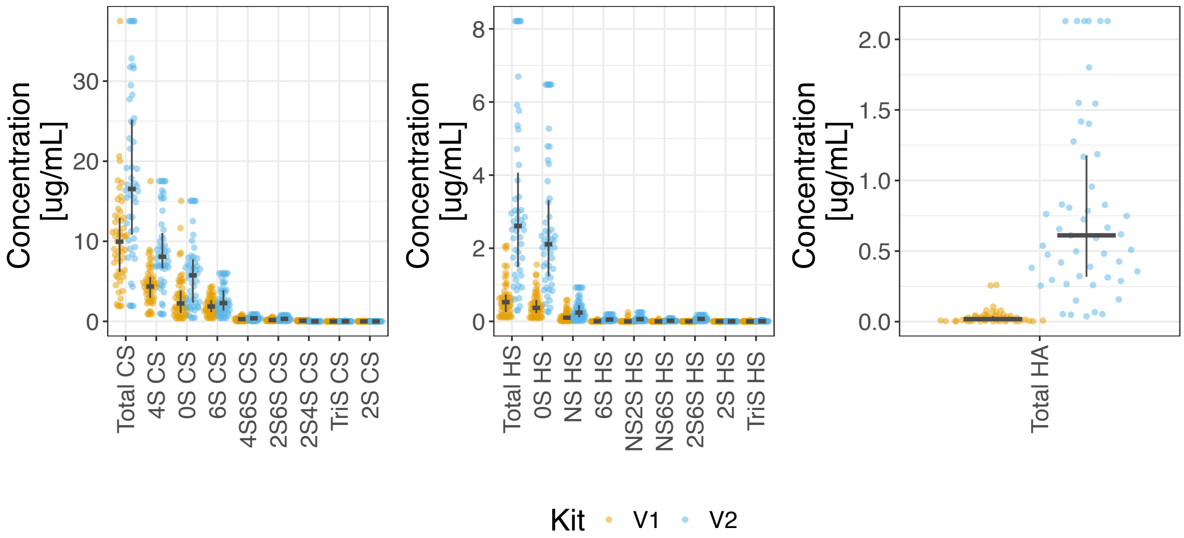

Supplementary Figure 3. Urine free GAGome in BCa patients from the development study (N = 51) using first generation (V1) vs. second generation (V2) kits. The concentrations were displayed with 90% winsorization. Key: CS – chondroitin sulfate; HS – heparan sulfate; HA – hyaluronic acid.

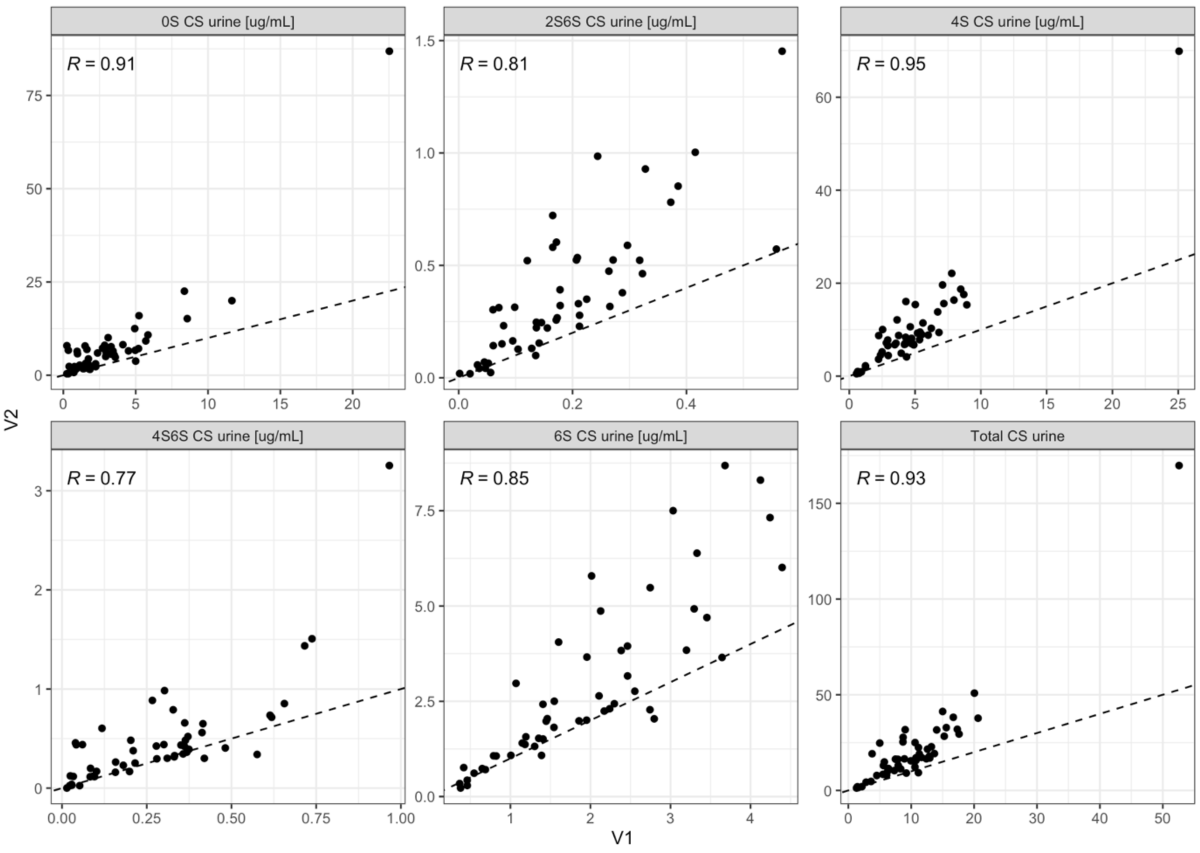

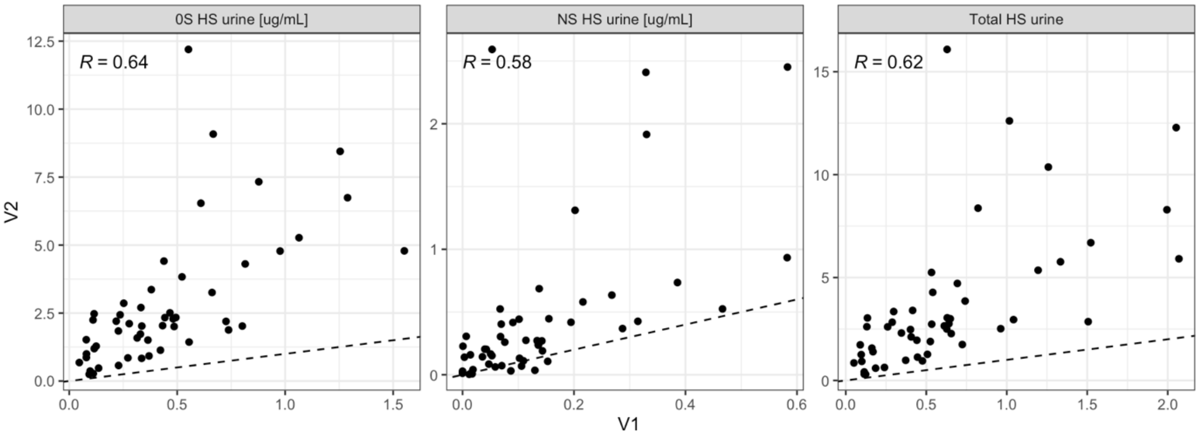

Supplementary Figure 4. Comparison between the concentration of each detectable urine free GAGome feature as measured in BCa patients from the development study (N = 51) using first generation (V1) vs. second generation (V2) kits. *R* indicates the Pearson correlation coefficient between the two scores ($p < 0.0001$ for all correlations). Key: CS – chondroitin sulfate; HS – heparan sulfate; HA – hyaluronic acid.

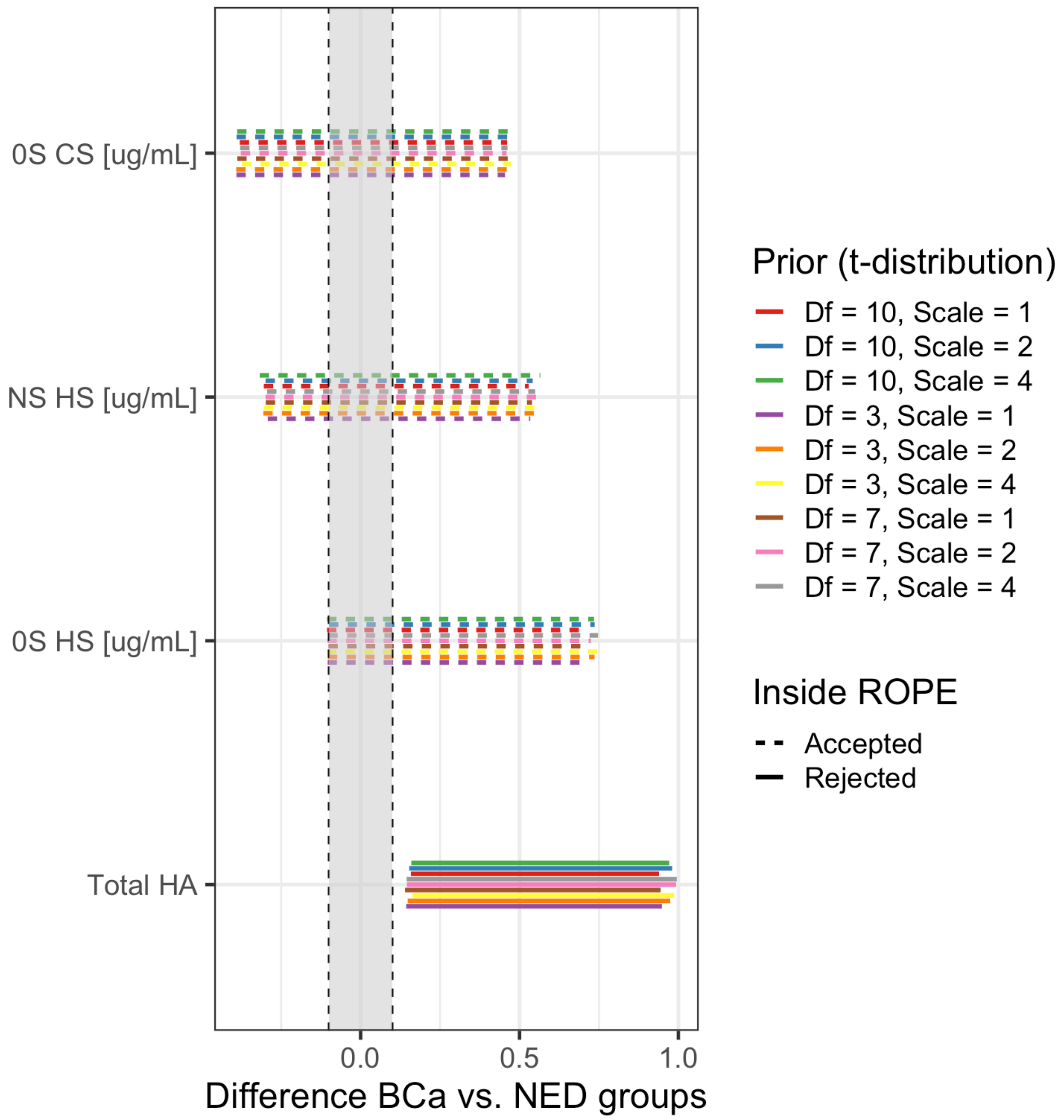

Supplementary Figure 5. Analysis of sensitivity of estimates of group differences to the parameters of the prior distribution in the development study. The points correspond to estimated parameter medians and the lines show the 95% credibility interval. The region of practical equivalence (ROPE) was marked by the two vertical dashed lines. Key: BCa – bladder cancer, Df – degrees of freedom; NED – no evidence of disease, ROPE - Region of Practical Equivalence; CS – chondroitin sulfate; HS – heparan sulfate; HA – hyaluronic acid.

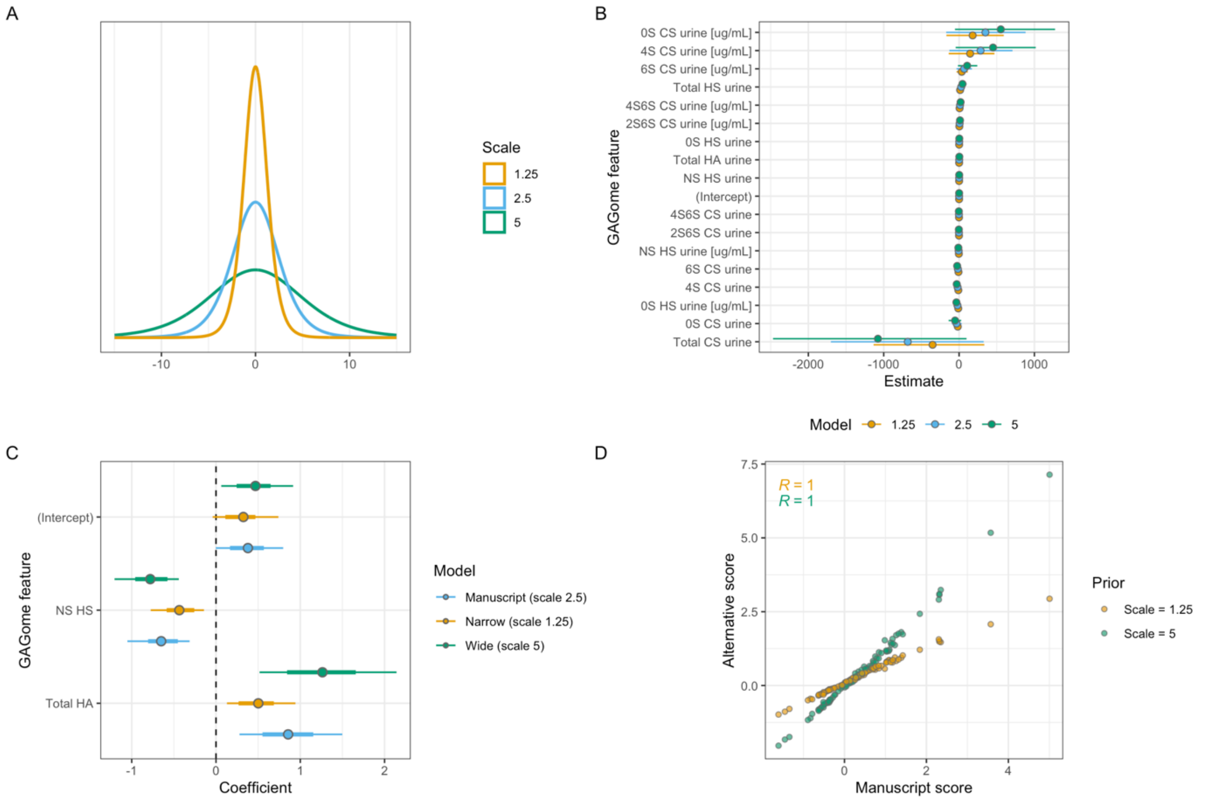

Supplementary Figure 6. Sensitivity analysis of the variable selection procedure for the urine free GAGome BCa score in the development study. The model described in the main text is fit using prior width 2.5 (blue in panels A-C). A) t-distribution priors with different priors (scale factors) used for to estimate standardized coefficients. B) Estimated coefficients in the reference model using the three different priors (note: GAGome features in the y-axis without "[ug/ml]" in the label are measured as mass fractions, %). C) Coefficients associated with selected variables resulting from alternative priors. D) Score correlation between the reference score (x-axis, described in the main text) and the score derived from an alternative prior (y-axis). R indicates the Pearson correlation coefficient between the two scores, $p < 0.0001$ for both correlations. Key: CS – chondroitin sulfate; HS – heparan sulfate, HA – hyaluronic acid.
